# A Higher Activation Threshold of Memory CD8^+^ T Cells Has a Fitness Cost That Is Modified by TCR Affinity during Tuberculosis

**DOI:** 10.1371/journal.ppat.1005380

**Published:** 2016-01-08

**Authors:** Stephen M. Carpenter, Cláudio Nunes-Alves, Matthew G. Booty, Sing Sing Way, Samuel M. Behar

**Affiliations:** 1 Department of Microbiology and Physiological Systems, University of Massachusetts Medical School, Worcester, Massachusetts, United States of America; 2 Division of Infectious Disease, Department of Medicine, Brigham and Women’s Hospital, Boston, Massachusetts, United States of America; 3 Division of Infectious Disease and Immunology, Department of Medicine, University of Massachusetts Medical School, Worcester, Massachusetts, United States of America; 4 Life and Health Sciences Research Institute (ICVS), School of Health Sciences, University of Minho, Braga, Portugal; 5 ICVS/3B’s—PT Government Associate Laboratory, Braga/Guimarães, Portugal; 6 Program in Immunology, Harvard Medical School, Boston, Massachusetts, United States of America; 7 Division of Infectious Diseases, Cincinnati Children’s Hospital, Cincinnati, Ohio, United States of America; New Jersey Medical School, UNITED STATES

## Abstract

T cell vaccines against *Mycobacterium tuberculosis* (Mtb) and other pathogens are based on the principle that memory T cells rapidly generate effector responses upon challenge, leading to pathogen clearance. Despite eliciting a robust memory CD8^+^ T cell response to the immunodominant Mtb antigen TB10.4 (EsxH), we find the increased frequency of TB10.4-specific CD8^+^ T cells conferred by vaccination to be short-lived after Mtb challenge. To compare memory and naïve CD8^+^ T cell function during their response to Mtb, we track their expansions using TB10.4-specific retrogenic CD8^+^ T cells. We find that the primary (naïve) response outnumbers the secondary (memory) response during Mtb challenge, an effect moderated by increased TCR affinity. To determine whether the expansion of polyclonal memory T cells is restrained following Mtb challenge, we used TCRβ deep sequencing to track TB10.4-specific CD8^+^ T cells after vaccination and subsequent challenge in intact mice. Successful memory T cells, defined by their clonal expansion after Mtb challenge, express similar CDR3β sequences suggesting TCR selection by antigen. Thus, both TCR-dependent and -independent factors affect the fitness of memory CD8^+^ responses. The impaired expansion of the majority of memory T cell clonotypes may explain why some TB vaccines have not provided better protection.

## Author Summary

CD8^+^ T cells are important for enforcing latency of tuberculosis, and for Mtb control in patients with HIV and low CD4 counts. While vaccines that primarily elicit CD4^+^ T cell responses have had difficulty preventing active pulmonary TB, a TB vaccine that elicits a potent memory CD8^+^ T cells is a logical alternative strategy. Memory T cells are thought to respond more rapidly than the primary (naïve) response. However, by directly comparing naïve and memory TCR retrogenic CD8^+^ T cells specific for the TB10.4 antigen during infection, we observe memory-derived T cells to be less fit than naïve-derived T cells. We relate the reduced fitness of memory CD8^+^ T cells to their lower sensitivity to antigen and show that fitness can be improved by increasing TCR affinity. Using a novel method for tracking CD8^+^ T cells elicited by vaccination during the response to Mtb aerosol challenge in intact mice, we observe the robust expansion of a new primary response as well as clonal selection of the secondary response, likely driven by TCR affinity. We propose that generating memory T cells with high affinities should be a goal of vaccination against TB.

## Introduction

The goal of T cell vaccination is to establish pre-existing immunity against pathogens in the form of memory T cells. Two features of memory T cells make them superior to naïve T cells in mediating protection. First, memory T cells have undergone a cycle of expansion and contraction, leading to a greater frequency of pathogen-specific T cells than present among the naïve T cell repertoire. Second, memory T cells do not classically require “priming” and rapidly express effector activity after antigen recognition, even after presentation by non-professional APCs. These features, together with their long-lived nature and their ability to survey non-lymphoid organs allow memory T cells to quickly respond to acute infection [1–5]. Although we have some understanding of the fate of memory T cell responses against pathogens that cause chronic infections, such as LCMV clone-13, a model pathogen that causes chronic infection in mice [6,7], little is known about the relative contribution of naïve and memory T cells (and the resulting 1° and 2° responses, respectively) to the recall response elicited by the human pathogen *Mycobacterium tuberculosis* (Mtb).

A vaccine that prevents tuberculosis is urgently needed but development efforts have been unsuccessful to date. The only approved vaccine, BCG, derived from an attenuated strain of *M*. *bovis*, is unable to confer reliable and long-lasting immunity in adults [8,9]. Similarly, Mtb vaccines show little evidence of long-lived protection in mouse models [10] despite 5–10 fold reductions in bacterial burden early during infection [11]. These results imply an early but transient benefit to memory T cells. Furthermore, despite the development of memory T cells following the successful treatment of active tuberculosis, observations of people cured by antibiotics show that they are not protected from reinfection [12–14]. Natural immunity to Mtb can be modeled in mice by clearing primary infection with antibiotics [15]. Upon re-challenge with aerosolized Mtb, a 10-fold CFU reduction is observed but the host remains chronically-infected. Furthermore, protection is short-lived and is accompanied by little or no change in survival [15,16]. These data highlight the need for better determinants of protective memory T cells.

CD8^+^ T cells are essential for the optimal control of *M*. *tuberculosis* [17–21]. Although the survival impact on mice depleted of CD8^+^ T cells is more modest than CD4^+^ T cell depletion during Mtb infection [20], we do not yet know which T cell subsets or functions are most important for a protective TB vaccine. CD8^+^ T cells are prime vaccine candidates for the prevention of disease since they are already believed to enforce latency in humans [22,23], and play a larger role in protective immunity in non-human primate models [21] compared with mouse models of infection. CD8^+^ T cells are able to directly kill infected cells and secrete cytokines in response to antigen presented by class I MHC, and can do so in cell types other than professional APCs such as lung epithelial cells [24]. Furthermore, CD8^+^ T cell responses are an important measure of the protective capacity of new vaccines in clinical trials [25,26]. The recent lack of protection found in clinical trials using MVA85A or M. bovis *BCG* vaccines, primarily eliciting CD4^+^ T cell responses, highlights our need to consider the importance of alternate T cell subsets and antigens in vaccine design [10,17,27].

TB10.4 (EsxH, Rv0288) is an immunodominant Mtb antigen recognized by human [28] and murine [29] CD8^+^ T cells. TB10.4 is already being tested in clinical trials [30] as a TB vaccine candidate since it is an essential gene and a secreted protein antigen of Mtb [31]. TB10.4-specific CD8^+^ T cells account for 30–50% of all CD8^+^ T cells in the lungs of infected C57BL/6 and BALB/c mice. TB10.4-specific CD8^+^ T cells can confer protection against Mtb after adoptive transfer into mice lacking αβ T cells [32], indicating that cells of this antigen specificity have the ability to attenuate bacterial growth. Although the protective capacity of antigen-specific CD8^+^ T cells is difficult to distinguish among the dominant CD4^+^ T cell response in intact mice, T cell vaccination that elicited a robust TB10.4-specific CD8^+^ T cell response did not protect mice from tuberculosis [33]. We find that TB10.4-specific memory CD8^+^ T cells elicited by vaccination undergo early and robust expansion after aerosol Mtb challenge, however the number of TB10.4-specific CD8^+^ T cells is similar to that of unvaccinated mice within four weeks. Using a combination of adoptive transfer of TCR retrogenic CD8^+^ T cells specific for TB10.4_4−11_ (TB10Rg3 and TB10Rg4) [32] and TCRβ deep sequencing of tetramer^+^CD8^+^ T cells after vaccination and Mtb-challenge in intact mice, we track primary (1°, those expanding from naïve T cells) and secondary (2°, from memory precursors) TB10.4-specific CD8^+^ T cells during infection. When naïve and memory CD8^+^ T cells expressing the same TCR are compared, we observe that both the 1° (naïve) and the 2° (memory) CD8^+^ T cell responses are initiated in the draining lymph node at ~d11 post-infection. Following the activation of the TB10.4-specifc CD8^+^ T cells, the 2° effector response does not rapidly expand in response to infection, but initially has the same kinetics as the 1° response. As the T cells are recruited to the lung, we also observe the 2° response becomes outnumbered 99:1 by a highly-proliferative 1° response, indicating that TCR-independent factors cause the memory-recall response to be less fit than the primary response during chronic infection. Using TCRβ deep sequencing, we find enormous clonotypic diversity in the TB10.4-specific CD8^+^ T cell response to vaccination, but after Mtb challenge the 2° response undergoes selection for a specific TCR motif that we attribute to higher affinity. By comparing the response of two different TCRs that differ in their affinity for the same epitope, we show that memory-derived CD8^+^ T cells with an increased affinity for antigen have greater fitness, demonstrating that TCR-dependent factors promote successful continued expansion of 2° effector CD8^+^ T cell responses during chronic infection in the lung. As we observe 2° effector CD8^+^ T cells to have a reduced proliferative response, particularly in chronic, low antigen settings, we speculate that effective T cell vaccines for tuberculosis will need to elicit high affinity TCRs and respond earlier than the primary response to infection.

## Results

### Vaccination elicits TB10-specific memory CD8^+^ T cells that expand after Mtb challenge

**Fig 1:**
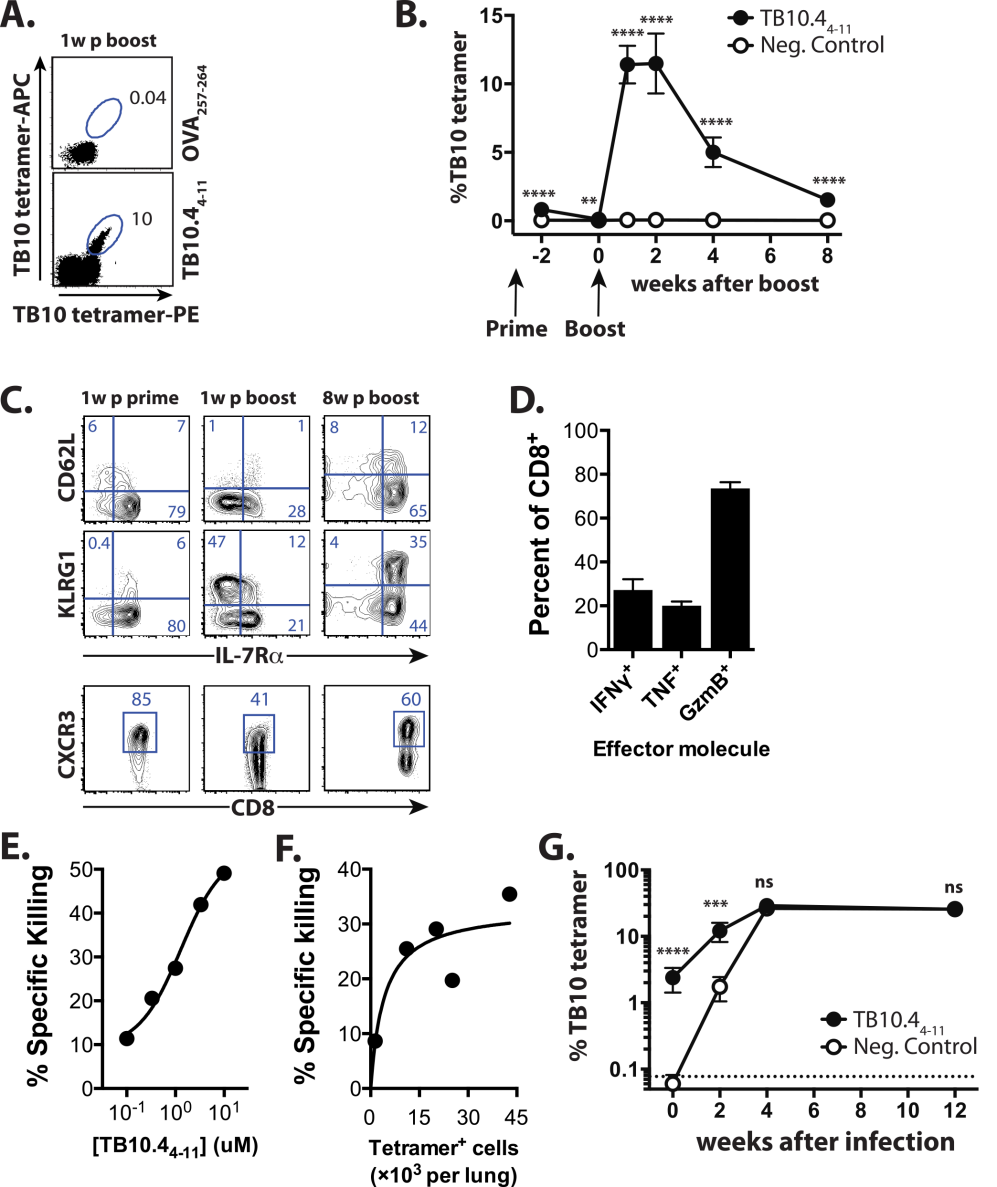
TB10 vaccination elicits memory CD8^+^ T cells that generate 2° effectors during Mtb infection. **(a)** The TB10 tetramer^+^ response enumerated by duel-tetramer staining in blood 1 week post-boost with TB10_4-11_ or Ova_257-264_ (control) vaccination. Numbers indicate the % of CD8^+^ T cells. **(b)** TB10 tetramer^+^ responses from blood after TB10_4-11_ or control (B8R_20-27_ or Ova_257-264_) vaccination at time points post-boost. **(c)** Representative plots showing CD62L, KLRG1, IL-7R, and CXCR3 expression by TB10 tetramer^+^ CD8^+^ T cells from blood 1w after prime, and 1w or 8w after boost. (d) Ex vivo TB10_4-11_-stimulated production of IFNγ, TNF, or granzyme B from CD8^+^ T cells isolated from combined lungs, spleens, or LNs of TB10-vaccinated mice. **(e)** In vivo specific killing of targets coated with TB10_4-11_ peptide. **(f)** In vivo specific killing of 1μM TB10_4-11_-coated targets vs. TB10-specific response. **(g)** TB10 tetramer responses of mice vaccinated with TB10_4-11_ or the control peptide B8R_20-27_ immediately prior, or 2w, 4w, or 12w after Mtb infection. **** p < 0.0001, *** p < 0.001, by two-way ANOVA with Sidak’s post test. Data are representative of 3–6 independent experiments, each with 4–6 mice per group.

Vaccination with the peptide epitope TB10.4_4−11_ (TB10), anti-CD40 mAb and poly(I:C), a vaccination strategy used in multiple infection and tumor models [34–37], generates a large number of TB10-specific memory CD8^+^ T cells in C57BL/6 mice. Boosting leads to an additional 10-fold expansion such that TB10-specific CD8^+^ T cells comprise ~10% of circulating CD8^+^ T cells (Fig 1A). Eight weeks after boosting, 1.5–2% of the circulating CD8^+^ T cells are specific for TB10 (Fig 1B). As described, this vaccine formulation represents a powerful and simple strategy to elicit high-frequency memory CD8^+^ T cell responses to multiple different tumor and viral epitopes under inflammatory conditions, and the T cells it generates are potent CTLs shown to eradicate melanoma lung metastases, and lower viral loads in both Ebola and RSV model infections [34–37]. The TB10 tetramer^+^CD8^+^ T cells elicited one week after priming are predominantly KLRG1^lo^IL-7R^hi^, the phenotype of memory precursor effector cells (MPECs) [38,39]. After boosting, ~50% express KLRG1 but low IL-7R levels, characteristic of terminally-differentiated effectors (Fig 1C), with early effector cells [40] (EECs, KLRG1^lo^IL-7R^lo^) and MPECs each comprising ~20% of TB10 tetramer^+^CD8^+^ T cells. Eight weeks later, TB10-specific CD8^+^ T cells are predominantly IL-7R^hi^ and ~50% express CD62L (Fig 1C). The majority of the TB10-specific CD8^+^ T cells express CXCR3, a chemokine receptor associated with CD27/CD70-dependent clonal expansion during priming [41], as well as trafficking of memory T cells to the airway during inflammation [42] (Fig 1C). Thus, the TB10/CD40/poly(I:C) vaccination strategy elicits large numbers of TB10-specific central memory and effector memory CD8^+^ T cells.

TB10-specific memory CD8^+^ T cells produced IFNγ, TNF, and granzyme B after *ex vivo* restimulation with the TB10 peptide (Fig 1D). Vaccine-elicited TB10-specific CD8^+^ T cells also efficiently lysed peptide-loaded targets *in vivo* in a dose-dependent manner (Fig 1E and 1F). These data show that the effector functions expressed by vaccine-elicited CD8^+^ T cells are similar to those possessed by CD8^+^ T elicited by Mtb infection [43]. To determine how these memory T cells respond to infection, we vaccinated with TB10 or an irrelevant peptide (B8R_20-27_ from vaccinia [44]) and eight weeks after the boost, infected the mice with Mtb. A discrete population of TB10-specific CD8^+^ T cells was detected in the lungs of the TB10 vaccinated mice even before infection (Fig 1G). Two weeks after Mtb challenge, TB10-specific CD8^+^ T cells were more frequent in the lungs of TB10-vaccinated mice compared to the B8R-vaccinated group, although there was no difference in the number of tetramer^+^ cells between the two groups by four weeks (Fig 1G). Thus, by the peak of adaptive immunity in C57BL/6 mice (4 wpi), there was no difference in the number of TB10-specific CD8^+^ T cells in the lungs of vaccinated and control-vaccinated mice despite the effectiveness of TB10/CD40/poly(I:C) vaccination in eliciting numerous functional memory CD8^+^ T cells.

**Fig 2:**
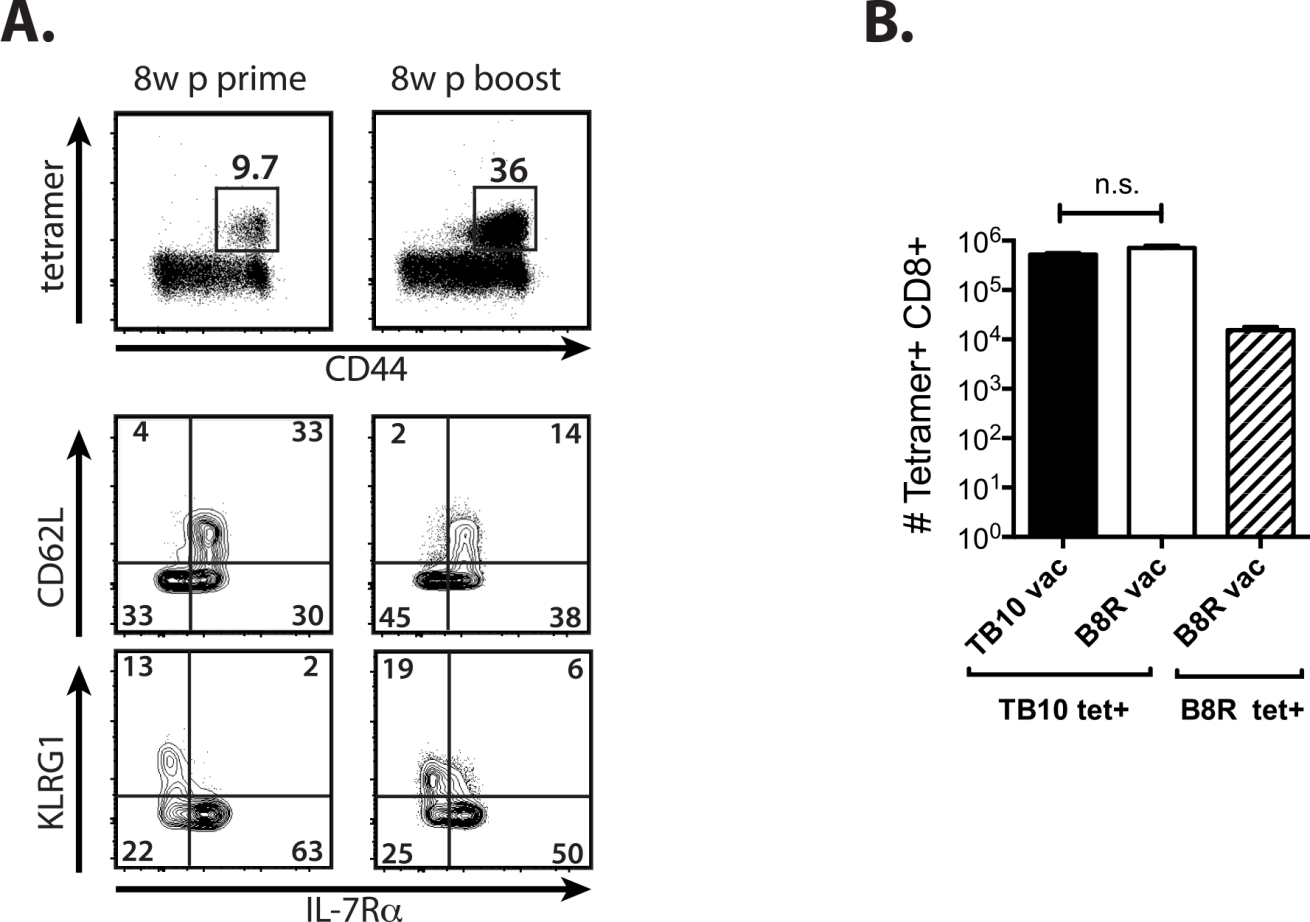
Vaccination with an amphiphilic TB10 peptide increases the precursor frequency but does not improve the kinetics the recall response. **(a)** Peripheral blood TB10 tetramer^+^ responses 8w post-prime or 8w post-boost with amphi-TB10 vaccination. CD62L, KLRG1, and IL-7R expression by tetramer^+^ CD8^+^ T cells is shown for each time point. **(b)** Lung tetramer^+^ responses from amphi-TB10 prime/boost-vaccinated (3 weeks apart), negative control-vaccinated (B8R), or unvaccinated mice 28d after Mtb infection. **** p < 0.0001, *** p < 0.001, n.s. not significant by one-way or two-way ANOVA with Sidak’s post test. Data are representative of 2–3 independent experiments, each with 4–6 mice per group.

Although vaccine-elicited TB10-specific CD8^+^ T cells were potent effectors and expanded early during infection, no differences were detected in the bacterial burden of vaccinated versus control mice (S1 Fig). We next sought to determine whether the lack of protection was related to insufficient numbers of memory CD8^+^ T cells prior to infection. As lipophilic vaccine adjuvants increase antigenicity [45], we modified the TB10 epitope by adding the hydrophobic amino acid residues ‘MFVMFVQ’ to the N-terminus of the minimal epitope of TB10. Eight weeks after priming and boosting with this amphiphilic peptide, denoted here as amphi-TB10, a greater proportion (20–45%) of circulating CD8^+^ T cells were specific for TB10, the majority of which were MPECs (Fig 2A). Despite a more robust response, neither prime-only nor prime-boost vaccination with amphi-TB10 increased antigen-specific CD8^+^ T cell frequency or attenuated bacterial growth compared to sham-vaccinated mice by 4wpi (Fig 2B, S1 Fig).

Thus, memory TB10-specific CD8^+^ T cells elicited by peptide/anti-CD40/poly (I:C) vaccination are highly-functional as measured by their expression of IFNγ, TNF, and granzyme B after stimulation, their CTL activity, their abundance 8–12 weeks after the boost, and their response to Mtb aerosol challenge. Together, these data raise the possibility that the inability of 2° effector CD8^+^ T cells to predominate in response to Mtb is related to a failure of memory CD8^+^ T cells to optimally expand rather than insufficient numbers prior to infection.

### Direct comparison of memory and naïve T cells using TCR retrogenic TB10-specific CD8^+^ T cells

**Fig 3:**
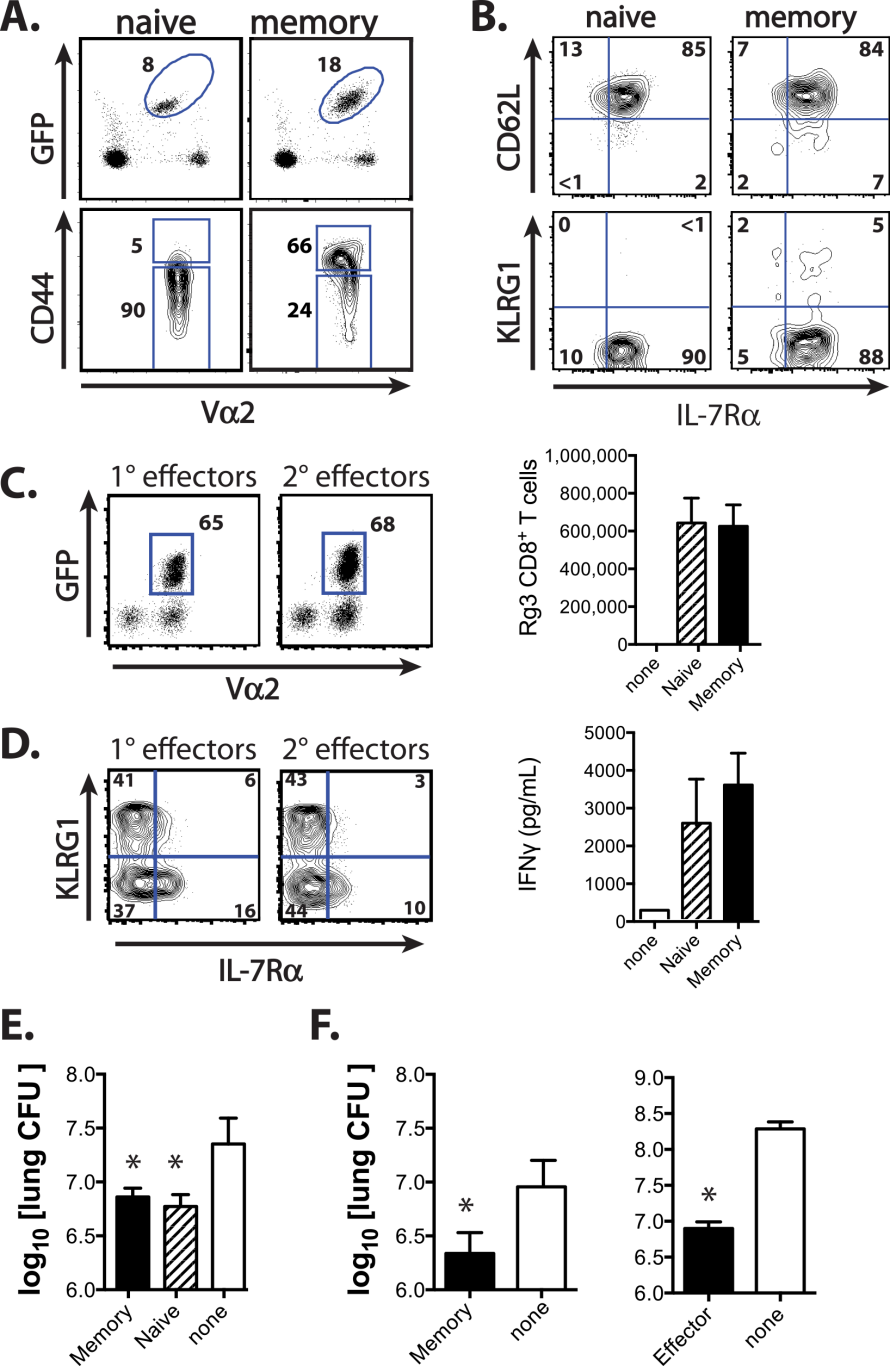
TCR retrogenic TB10-specific CD8^+^ T cells allow direct comparison of the 1° and 2° responses during infection. **(a)** The proportion of TB10Rg3 cells (%GFP^+^Vα2^+^) among CD8^+^ T cells and their CD44 expression, 8w after vaccination of TCR Rg mice (memory) or age-matched unvaccinated TCR Rg mice (naïve). **(b)** CD62L, KLRG1, and IL-7R expression of naïve TB10Rg3 cells, and 8w after a single immunization with TB10_4-11_. **(c)** Proportion (left) and absolute numbers (right) of TB10Rg3 cells in the lungs of TCRα^-/-^ mice 28d after adoptive transfer of 10^5^ naïve or memory TB10Rg3 CD8^+^ T cells and Mtb infection. **(d)** *Ex vivo* KLRG1 and IL-7R expression by TB10Rg3 cells (left) and IFNγ production after *ex vivo* stimulation of lung cells [from (**c**)] with TB10_4-11_ peptide (right). **(e)** Lung CFU of TCRα^-/-^ mice 28d after transfer of naïve or memory TB10Rg3 CD8^+^ T cells and Mtb infection. **(f)** Lung CFU of sub-lethally irradiated C57BL/6 mice 21d after Mtb infection and transfer of memory TB10Rg3 (8w post-vaccination; left) or effector TB10Rg3 (1w post-vaccination; right). Numbers in quadrants or gated regions represent percent events. CFU were log_10_-transformed before a student’s t-test or one-way ANOVA with a Bonferroni post-test. Data are representative of 2–4 independent experiments, each with 5 mice per group. * p < 0.05. n.s., not significant.

To directly compare how memory and naïve TB10-specific CD8^+^ T cells behave during Mtb challenge, we used TCR retrogenic (Rg) mice producing CD8^+^ T cells specific for TB10 (TB10Rg3) [32]. Vaccination increased the frequency of TB10Rg3 CD8^+^ T cells (GFP^+^Vα2^+^), and 60–70% of expressed CD44 compared with ≤5% in unvaccinated mice (Fig 3A). After 8 weeks, TB10Rg3 cells contracted into a uniform population of central memory T cells (CD62L^hi^IL-7R^hi^) (Fig 3B).

We previously found that the adoptive transfer of activated TB10Rg3 CD8^+^ T cells reduced bacterial CFU and prolonged the survival of susceptible mice [32]. Here we compared the protective capacity of flow-sorted naïve (GFP^+^Vα2^+^CD44^lo^ cells) or memory (GFP^+^Vα2^+^CD44^Hi^) TB10Rg3 CD8^+^ T cells by transferring 10^5^ of each into TCRα^-/-^ mice and challenging with Mtb. Both naïve and memory TB10Rg3 CD8^+^ T cells expanded and differentiated into terminally-differentiated effectors (KLRG1^Hi^IL-7R^lo^) and EECs (KLRG1^Lo^IL-7R^lo^), with a small population of MPECs (KLRG1^Lo^IL7R^Hi^), and produced IFNγ after restimulation in vitro (Fig 3C and 3D). Naïve, effector, and memory TB10Rg3 CD8^+^ T cells transferred protection to immunodeficient mice (Fig 3E and 3F), indicating that these cells have the potential to independently function as effector T cells and attenuate infection.

Although memory TB10Rg3 CD8^+^ T cells expanded, differentiated, and attenuated bacterial growth after adoptive transfer, the inability to distinguish the memory-recall response in intact vaccinated mice from the primary response in unvaccinated mice by 4wpi (Fig 1G), as well as the inability of TB10 vaccination to confer additional protection to the endogenous primary immune response [33] (S1 Fig), led us to hypothesize that memory CD8^+^ T cells were not optimally responding in vivo. In our comparison of TB10-specific CD8^+^ T cell responses of vaccinated and unvaccinated mice, the primary response (e.g., in unvaccinated mice) appears to undergo more rapid expansion than the recall response (e.g., in vaccinated mice) (Fig 1G). Therefore, we developed an adoptive co-transfer model to study the 1° and 2° effector responses generated from naïve and memory TB10Rg3 CD8^+^ T cells, respectively, during Mtb challenge.

### Primary effectors progressively outnumber secondary effector CD8^+^ T cells during Mtb infection

**Fig 4:**
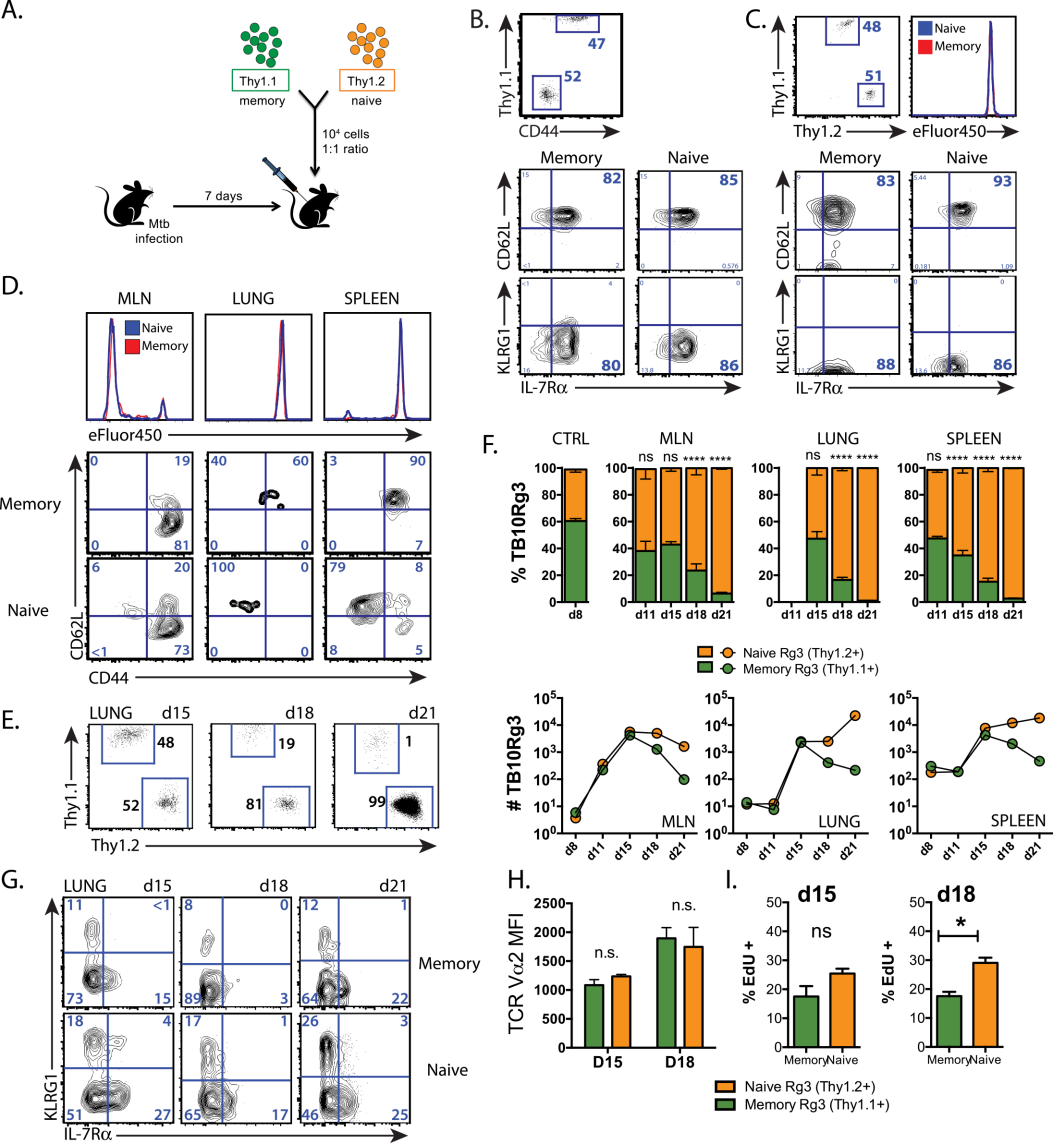
The primary response dominates the memory-derived secondary response during Mtb infection. **(a)** Experimental strategy for adoptive co-transfer experiments. Relative proportion of naïve and memory TB10Rg3 CD8^+^ T cells and their expression of CD62L, KLRG1, and IL-7R before transfer **(b)** and in the spleen 1d after transfer into uninfected mice **(c)**. Baseline labeling with the eFluor450 proliferation dye is shown. **(d)** Concatenated histograms of eFluor450 staining of naïve and memory-derived TB10Rg3 cells in the MLN, lung, and spleen (top) and their CD62L and CD44 expression (bottom) from a representative experiment on d11 post-infection. **(e)** Proportion of adoptively-transferred memory (Thy1.1^+^) and naïve (Thy1.2^+^)-derived TB10Rg3 CD8^+^ T cells in the lung 15, 18, or 21d after Mtb infection. **(f)** The relative proportion of memory (Thy1.1) and naïve (Thy1.2)-derived TB10Rg3 CD8^+^ T cells in the MLN, lung, and spleen after infection, compared to spleens from uninfected mice 1 day after transfer (CTRL) (top). Cell numbers of memory (Thy1.1^+^) and naïve (Thy1.2^+^)-derived TB10Rg3 CD8^+^ T cells from the same mice (bottom). (**g**) KLRG1 and IL-7R expression by memory and naïve-derived TB10Rg3 cells recovered from lung at each time point. **(h)** TCR Vα2 median fluorescence intensity (MFI) (median ± SEM) in memory and naïve-derived TB10Rg3 CD8^+^ T cells from the same mice at d15 and d18 post-infection. **(i)** EdU uptake (mean ± SEM) by memory and naïve-derived TB10Rg3 cells recovered from lung. EdU uptake was compared with a student’s t-test. * p < 0.05, ** p < 0.01, *** p < 0.001, **** p < 0.0001 n.s. not significant, n.d. < 10 cells detected. Data are representative of 2–10 independent experiments, each with 3–4 mice per group.

Thy1.1^+^ memory and Thy1.2^+^ naïve TB10Rg3 CD8^+^ T cells were transferred (1:1 ratio, 10^4^ cells each) into CD45.1^+^ recipient mice infected with Mtb seven days earlier, before the onset of adaptive immunity (Fig 4A and 4B). Nearly all (>80%) of the memory TB10Rg3 CD8^+^ T cells had a central memory phenotype (CD62L^hi^KLRG1^lo^IL-7R^hi^), suggestive of a high proliferative potential (Fig 4B). As a control, the memory and naïve TB10Rg3 CD8^+^ T cells were adoptively transferred into uninfected or Mtb-infected mice and analyzed the next day. Analysis of these cells showed that they maintained their phenotype, 1:1 ratio, and based on results with the eFluor450 proliferation dye, neither group had begun to proliferate (Fig 4C).

While naïve T cells require priming in the lung-draining mediastinal lymph node (MLN) before responding to infection in the lung [46], whether memory T cell activation occurs in the MLN or lung is unknown. By loading naïve and memory TB10Rg3 CD8^+^ T cells with eFluor450, we determined that both memory and naïve TB10Rg3 CD8^+^ T cells begin to proliferate and downregulate CD62L first in the MLN by d11 post-infection (Fig 4D), during which time they maintain their 1:1 ratio despite significant proliferation (see below). This timing correlates with priming of the endogenous CD8^+^ T cell response to TB10 [32].

Following activation in the MLN, massive expansion of naïve and memory TB10Rg3 CD8^+^ T cells occurs in the MLN, lung and spleen through day 15, still maintaining an equal ratio (Fig 4E and 4F). After day 15, the 1° effectors (derived from naïve T cells) become dominant in all three tissues, and by day 21, the 1° effectors outnumber the 2° effectors (derived from memory T cells) by a ratio of 99:1 in the lung (Fig 4E and 4F). The accumulation of the 1° effectors is driven by their ongoing proliferation and dropout of 2° effector cells, particularly in the lung (Fig 4F, bottom row). Thus, the 1° CD8^+^ T cell response expands more efficiently than the 2° response during Mtb challenge.

### Sustained proliferation of primary effectors during infection leads to their dominance

Joshi et al. find that during infection, a subset of effector CD8^+^ T cells differentiate into cells that can no longer proliferate in response to antigen and express the inhibitory receptor KLRG1, now identified as a marker of terminal differentiation [39]. We examined whether the attrition of the secondary effectors correlated with terminal differentiation. Both 1° and 2° effector CD8^+^ T cells were predominantly EECs (KLRG1^Lo^IL-7R^Lo^) at all time points (day 15–21), with slightly more terminally-differentiated effectors (KLRG1^Hi^IL-7R^Lo^) in 1° effectors, rather than in 2° effectors (Fig 4G), arguing against terminal differentiation as an explanation for their observed decreased rate of expansion. The attrition of secondary effector CD8^+^ T cells during infection is also independent of TCR affinity, since TCR retrogenic TB10Rg3 cells were the source of both the naïve and memory precursors. Furthermore, we found TCR expression to be equivalent between both memory and naïve-derived TB10Rg3 cells during infection (Fig 4H). Finally, since Mtb-specific T cells can differ in their ability to traffic to the lung [47,48], we considered whether naïve and memory-derived TB10Rg3 cells might differentially home to the lung. Equal proportions of 1° and 2° effector CD8^+^ T cells were in the “intravascular” or “parenchymal” compartments, as defined by intravenous administration of anti-Vα2 mAb. Thus, the 1° and 2° effector CD8^+^ T cell responses were able to traffic similarly to the lungs of Mtb-infected hosts.

Next we determined whether 1° and 2° effector CD8^+^ T cells proliferate differently during infection. On d18 post-infection, 1° effector TB10Rg3 CD8^+^ T cells had ~40% more EdU uptake than 2° effectors (Fig 4I). In contrast, no differences in the frequency of apoptotic cells, measured using an activated caspase-3 antibody or with a viability dye, were detected (S2 Fig). These data suggest 2° effectors become outnumbered due to a decreased rate of 2° effector CD8^+^ T cell proliferation after d15, while a greater rate of 1° effector proliferation leads to continued exponential expansion.

### Memory CD8^+^ T cells have a higher activation threshold than naïve CD8^+^ T cells

**Fig 5:**
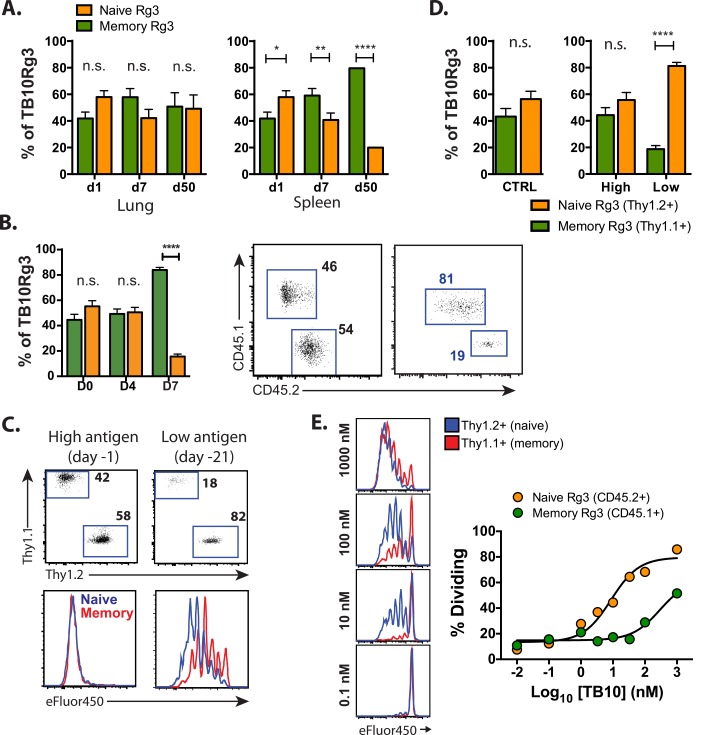
Memory CD8^+^ T cells proliferate but have a higher activation threshold than naïve CD8^+^ T cells. **(a)** The proportions of naïve and memory-derived TB10Rg3 cells in the lung (left) or spleen (right) 1, 7, or 50 d after adoptive co-transfer into mice administered amphi-TB10/poly(I:C)/CD40 1d prior to transfer. **(b)** Bar graphs (left) and representative flow plots (right) of the proportion of splenic TB10Rg3 CD8^+^ T cells (mean ± SEM) derived from memory (CD45.1^+^) or naïve (CD45.2^+^) TB10Rg3 0, 4, or 7 days after i.v. LmΔActA-TB10 challenge of Thy1.1^+^ hosts, in which 10^4^ memory and naïve TB10Rg3 cells were co-transferred at a 1:1 ratio 1 day prior to challenge. **(c, d)** The proportion of splenic TB10Rg3 cells (mean ± SEM) derived from naïve or memory TB10Rg3 3d after their co-transfer into mice administered amphi-TB10/poly(I:C)/CD40 1d (high antigen) or 21d (low antigen) prior to transfer. Their ratios and dilution of proliferation dye are shown. **(e)** eFluor450 dilution by naïve or memory TB10Rg3 cells 64h after culture with peptide-coated splenocytes (left) and summary of dose-response data (right). Memory: naïve T cell ratios across time points were compared by a 2-way ANOVA and the Bonferroni post-test. * p < 0.05, ** p < 0.01, **** p < 0.0001, n.s. not significant. Data are representative of 2–3 independent experiments, each with 3–4 mice per time point.

To determine whether the observed reduced proliferation was an intrinsic property of memory CD8^+^ T cells, or was precipitated by extrinsic signals in the inflammatory environment of the infected lung, we studied T cell expansion in a model of acute infection and two non-infectious models. First, naïve and memory TB10Rg3 CD8^+^ T cells were co-transferred into mice challenged with amphi-TB10 peptide together with anti-CD40 mAb and poly(I:C) one day earlier. One week after transfer into amphi-TB10 challenged mice, significant expansion had occurred in both groups but the ratio of 1° and 2° effectors remained ~1:1, with a predominance of 2° effectors late during expansion (Fig 5A). Homeostatic proliferation of naïve and memory TB10Rg3 CD8^+^ T cells was also measured three weeks after their transfer into TCRα^-/-^ mice and the dividing cells also maintained an equal ratio (S3 Fig). Finally, one day after 1:1 co-transfer of memory and naïve TB10Rg3 CD8^+^ T cells, mice were challenged intravenously with recombinant *Listeria monocytogenes* engineered to secrete a fusion protein containing full-length TB10.4 (LmΔActA-TB10) [49,50]. Four days after LmΔActA-TB10 challenge, during a period of robust expansion, both groups expanded equally to TB10.4 antigen. During the contraction of the response (d7), the TB10Rg3 CD8^+^ T cells derived from memory were more abundant than those derived from naïve TB10Rg3 CD8^+^ T cells resulting in an 80:20 ratio favoring the 2° effectors (Fig 5B). Thus, 2° effector CD8^+^ T cells have the potential to proliferate as well as 1° effectors during acute infection or after non-infectious antigenic stimuli.

Mehlhop-Williams and Bevan find that memory CD8^+^ T cells require more antigen for activation than naïve CD8^+^ T cells, which results in less proliferation of secondary effectors when antigen is limiting [51]. Both the antigen-challenge and the listeria models are scenarios in which TB10.4_4−11_ is likely to be present in abundance. To formally determine whether the proliferation of memory TB10Rg3 CD8^+^ T cells is affected by antigen availability, mice were challenged with amphi-TB10/αCD40/poly(I:C) i.v. 1d or 21d prior to co-transfer of naïve and memory TB10Rg3 CD8^+^ T cells to simulate high or low antigen conditions, respectively. Both naïve and memory CD8^+^ T cells proliferated similarly 3d after exposure to high antigen conditions (Fig 5C and 5D). However, 1° effectors underwent more cell divisions than 2° effectors when exposed to low antigen conditions (Fig 5E), and increased in number relative to the 2° effectors. Finally, naïve and memory TB10Rg3 CD8^+^ T cells differed in their sensitivity to peptide concentration in vitro. Memory T cells required 32-fold more peptide to trigger proliferation than naive T cells (Fig 5E). Thus, a low antigen environment recapitulates the bias towards primary effectors that we identified during Mtb infection.

### Higher TCR affinity offsets memory CD8^+^ T cell loss during tuberculosis

Since greater amounts of peptide-MHC complexes (pMHC) are required to trigger memory CD8^+^ T cell entry into the cell cycle, we determined whether TCR affinity modulated the fitness of memory CD8^+^ T cells during the response to Mtb infection. In addition to the TB10Rg3 mice, we recently generated TB10Rg4 retrogenic mice containing TB10-specific CD8^+^ T cells that use a TCR that has a higher affinity for TB10.4_4−11_ [32]. We vaccinated mice containing congenically-marked TB10Rg4 CD8^+^ T cells and rested age-matched, naive TB10Rg4 mice for an equivalent period of time (8–12 weeks) to compare the expansions of higher-affinity memory CD8^+^ T cells with their naïve counterparts.

**Fig 6:**
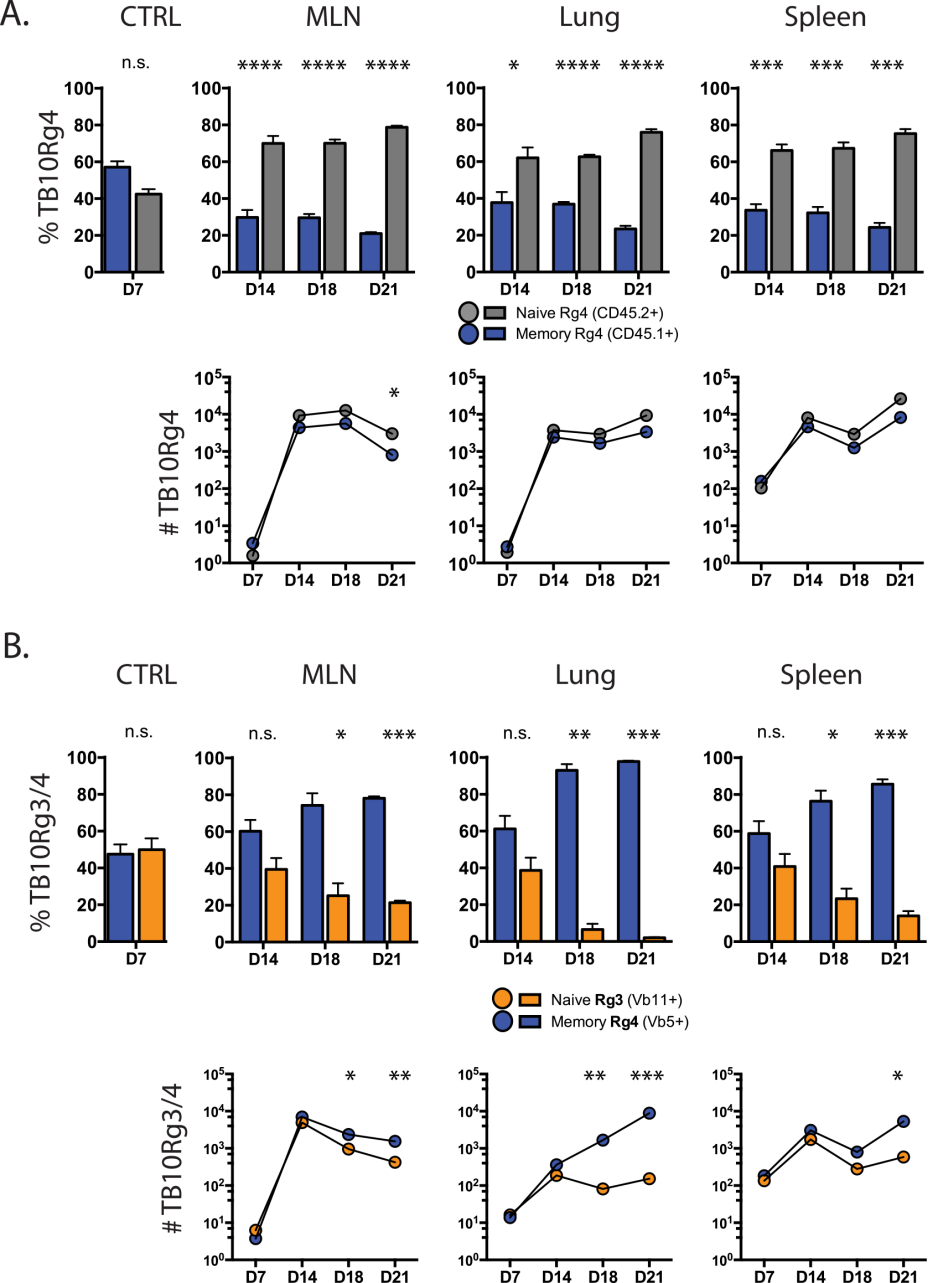
Memory CD8^+^ T cells with a higher affinity TCR can display improved responses during tuberculosis. **(a)** Proportion of adoptively-transferred memory (CD45.1^+^) and naïve (CD45.2^+^)-derived TB10Rg4 CD8^+^ T cells in the MLN, lung, and spleen 14, 18, or 21d after Mtb infection, compared to spleens from uninfected mice 1 day after adoptive transfer (CTRL) (top). Cell numbers of memory and naïve-derived TB10Rg4 CD8^+^ T cells from the same mice (bottom). **(b)** The relative proportion of adoptively-transferred, memory-derived TB10Rg4 (Vβ5^+^) and naïve-derived TB10Rg3 (Vβ11^+^) CD8^+^ T cells in the MLN, lung, and spleen 14, 18, or 21d after Mtb infection, compared to those in the spleens of uninfected mice 1 day after adoptive transfer (CTRL) (top). Cell numbers of memory-derived (TB10Rg4) and naïve-derived (TB10Rg3) CD8^+^ T cells from the same mice at each time point during infection (bottom). * p < 0.05, ** p < 0.01, *** p < 0.001, **** p < 0.0001, n.s. not significant. Data are representative of 2 independent experiments, each with 4 mice per group.

To determine whether the affinity of the TCR affects the relative ability of memory and naïve CD8^+^ T cells to expand, we co-transferred memory and naïve TB10Rg4 CD8^+^ T cells at a 1:1 ratio into Mtb-infected mice. Using the same methodology (Fig 4A), we tracked the relative expansions of 1° and 2° effector CD8^+^ T cells. Similar to our previous results, the TB10Rg4 naïve CD8^+^ T cells expanded more than TB10Rg4 memory CD8^+^ T cells (Fig 6A). Although those derived from TB10Rg4 memory were again outnumbered by d21 post-infection, the effect was less extreme, resulting in a ratio of ~4:1 favoring the 1° effector CD8^+^ T cells in MLN, lung, and spleen (Fig 6A). Differences in the expansion of memory and naïve TB10Rg4 CD8^+^ T cells were again independent of surface TCR levels as TCR Vα2 MFI were equivalent (S4 Fig). Thus, for a second TB10.4_4−11_–specific TCR, we see a similar predilection for the 1° effectors to outnumber the 2° effector CD8^+^ T cells. Although these higher-affinity memory CD8^+^ T cells did not begin responding to Mtb earlier than the lower affinity (TB10Rg3) memory response, they displayed improved fitness.

We next sought to determine whether higher affinity memory T cells were more fit than lower affinity naïve T cells. Memory TB10Rg4 and naive TB10Rg3 CD8^+^ T cells were co-transferred at a 1:1 ratio into mice infected 6–7 days earlier, and their expansion and ratio tracked through d21 post-infection. Memory TB10Rg4 CD8^+^ T cells successfully competed, significantly outnumbering naïve-derived TB10Rg3 CD8^+^ T cells by d14 post-infection in MLN, lung, and spleen (Fig 6B). By d21, the 2° TB10Rg4 effectors dominated the 1° TB10Rg3 effectors by a ratio of 50:1. Although memory CD8^+^ T cells have a higher antigen threshold for their activation, a higher TCR affinity for pMHC helps memory-derived CD8^+^ T cells compete with those derived from naïve CD8^+^ T cells during tuberculosis. We infer that affinity plays an important role in the success of memory-derived effector CD8^+^ T cells during TB.

### TCRβ deep sequencing distinguishes primary and secondary T cell responses

**Fig 7:**
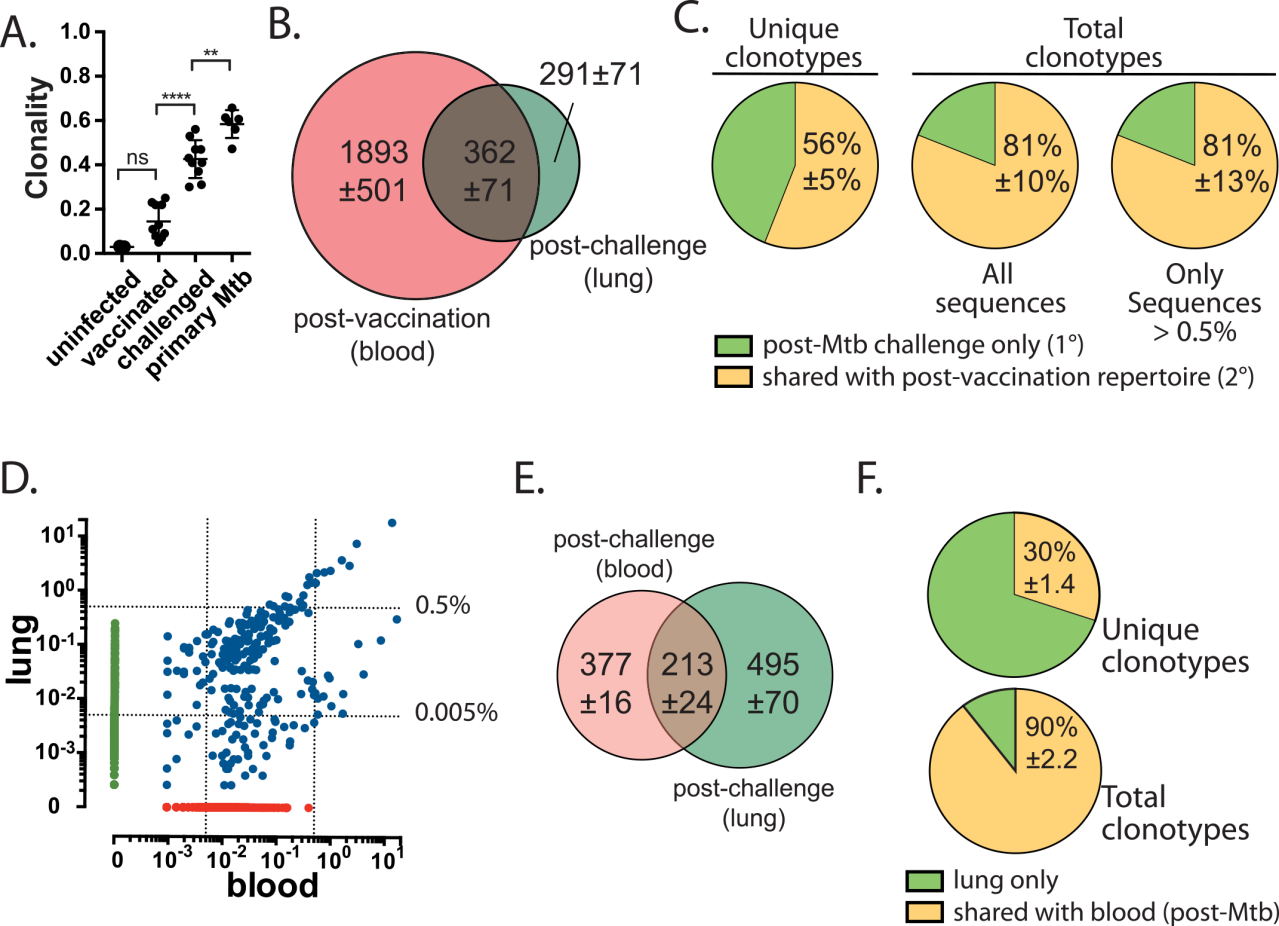
TCRβ deep sequencing reveals the dual contribution of the primary and secondary effector CD8^+^ T cell response in vaccinated mice challenged with Mtb. **(a)** Clonality of TB10-specific CD8^+^ T cells from blood 1w after amphi-TB10 vaccination, compared to those isolated from lung in the same individuals 4-5w after Mtb challenge, or compared to those isolated from unvaccinated, Mtb-infected mice [data for primary Mtb-infected mice from [32]]. Data are from 3–10 individuals/group, independently analyzed from two independent experiments. One-way ANOVA with a Bonferroni post-test was used to compare clonality. * p < 0.05, **** p < 0.0001. **(b)** Sharing of unique TCRβ DNA sequences between the post-vaccination (blood) and post-Mtb challenge (lung) repertoires of TB10-specific CD8^+^ T cells. Numbers are the average of unique TCRβ DNA clonotypes, determined for four subjects, each analyzed individually. **(c)** The percentage of the lung TB10-specific CD8^+^ TCRs detected either only post-Mtb challenge (e.g., 1° response); or, detected both post-vaccination and post-Mtb challenge (e.g., 2° response). Left, unique clonotypes; center, total TCRs; right, total TCRs that had a frequency of >0.5%. **(d)** Representative logarithmic scatter plot showing the frequencies of all clonotypes detected in the blood or in the lung of a single individual 5 weeks after Mtb challenge. Green, lung only; Red, blood only; Blue, Shared. The dotted lines indicate frequencies of 0.005% and 0.5%, respectively. **(e)** Sharing of unique TCRβ DNA sequences between blood and lung repertoires of TB10-specific CD8^+^ T cells in individual mice, 5 weeks after infection. Numbers are the average of unique TCRβ DNA clonotypes, determined for three subjects analyzed individually. **(f)** The percentage of the lung repertoire of TB10-specific CD8^+^ TCRs that were detected only in the lung (“lung only”) or detected in the blood and lung (“shared with blood”) after Mtb challenge. Left, unique clonotypes; right, total TCRs. Only clonotypes with a frequency of >0.005% were analyzed.

By adoptively transferring well-characterized naïve and memory TB10Rg3 CD8^+^ T cells at a 1:1 ratio, we showed that factors other than TCR affinity or abundance determined the increased fitness of the naïve T cell response. On the other hand, our experiments using TB10Rg4 CD8^+^ T cells, which recognize the same epitope as TB10Rg3, but with a higher affinity, indicate that increased affinity can offset the disadvantage in the expansion rate of memory CD8^+^ T cells leading to their dominance over lower-affinity naïve CD8^+^ T cells during Mtb challenge. In reality, there exists considerable variation in frequency and TCR affinity in the T cell repertoire, which could affect the success of individual clonotypes. To determine how Mtb infection affects the ability of TB10-specific memory CD8^+^ T cells to expand in mice with an intact and diverse immune repertoire, we used NexGen TCRβ sequencing to track the polyclonal TB10-specific CD8^+^ T cell response to vaccination, and the subsequent recall response after Mtb challenge in individual mice. We reasoned that clonotypes elicited by vaccination that were also detected after challenge represented 2° effectors. On the other hand, clonotypes detected only after challenge were more likely to be part of a new 1° response. We purified TB10-specific CD8^+^ T cells using tetramers by flow sorting after vaccination, and again after Mtb challenge in the same individual, and sequenced their TCRβ repertoire. We find that after vaccination, the clonality of TB10-specific CD8^+^ T cells was not statistically different than that of total T cells from the peripheral blood of uninfected B6 mice (Fig 7A). However, TB10-specific CD8^+^ T cells were significantly more diverse after vaccination than after Mtb challenge (Fig 7A and S5 Fig). Thus, the post-challenge TCRβ repertoire was more similar to what we observed following primary Mtb infection [32]. Interestingly, the TB10-specific CD8^+^ T cells appeared to be less clonal after Mtb infection in mice that were previously vaccinated [compare ‘challenged’ vs. ‘primary Mtb’; ‘primary Mtb’ data from [32]], raising the possibility that vaccination leads to a more diverse T cell response during infection.

One week after vaccination, we detected 2254 ± 509 unique clonotypes among peripheral blood TB10-specific CD8^+^ T cells (Fig 7B). Four weeks after Mtb challenge, the repertoire of pulmonary TB10-specific CD8^+^ T cells consisted of 652 ± 125 unique clonotypes. On average, 56% (362 ± 71) of the unique clonotypes in the lungs of infected mice were previously detected after vaccination, meeting our definition of 2° effector T cells. Thus, nearly half of the distinct clonotypes specific for TB10.4 in the lungs of vaccinated mice challenged with Mtb were part of a new 1° response. However, not all clonotypes were similarly represented in the lung. While the shared sequences, which we define as 2° effectors, accounted for 56% of the unique clonotypes, they added up to 81% of all TB10-specific CD8^+^ T cells in the lung. A possible bias in our analysis is that clonotypes detected in the lung might not be detected in blood. However, the peripheral blood samples in this cohort of mice were sequenced at the maximum depth available to mitigate this possible confounder. We have found the naïve precursor frequency of TB10-specific CD8^+^ T cells in C57BL/6 mice to be ~1 in 20,000 (0.005%) [32], whereas the frequency of memory TB10-specific CD8^+^ T cells 8–12 weeks after vaccination is ~1:10 (Fig 2A). Therefore, we focused on only the abundant clonotypes in the lung, using a threshold of 0.005% or 0.5%, and we found that 84% and 81% of the total TCRs were shared, which is similar to when clonotypes of all frequencies are analyzed (Fig 7C). Thus, in vaccinated mice subsequently challenged with Mtb, nearly half of the unique clonotypes in the lung represent a new 1° response and in aggregate they make up ~20% of the total TB10-specific CD8^+^ T cells in the lung. Since the TB10-specific response represents >30% of all CD8^+^ T cells in the lung by 4wpi, the new 1° response expanded from ~1 in 20,000 CD8^+^ T cells (in the naïve repertoire) to ~6% of the CD8^+^ T cells in the lung. Likewise, the 2° response expanded from ~1 in 10 CD8^+^ T cells (in the post-vaccination repertoire) to ~24% of the CD8^+^ T cells in the lung. Thus, the new 1° response underwent a ~1,200-fold expansion compared to a ~2.4-fold expansion for the 2° response.

### TCRβ deep sequencing identifies TB10-specific CD8^+^ T cell clonotypes that are shared between lung and blood

Our evaluation of the post-vaccination and post-Mtb challenge TCR repertoires in the same individual requires the comparison of T cells in blood (post-vaccine) to lung (post-challenge). To further validate this approach, we asked to what degree the TB10-specific repertoire in the blood and lung are related. TB10-specific CD8^+^ T cells (e.g., tetramer^+^) were simultaneously isolated from the peripheral blood and perfused lung of individual Mtb-challenged mice by flow sorting. Although unique clonotypes exist that are detected only in lung or blood, all of the abundant TCR clonotypes detected in the lung (defined as a frequency >0.5%) were also detected in blood (Fig 7D). In each of the mice, two distinct clusters of T cell clonotypes could be identified: each with a similar frequency in blood but significantly different frequencies in lung. Although lung parenchymal and lung intravascular pools of T cells were not formally distinguished in this experiment, T cells in these newly-defined compartments might also exhibit such clustering by frequency in the lung [48,52].

There was substantial overlap between the clonotypes detected in blood and in lung (Fig 7D). We detected 400–600 distinct DNA sequences among pulmonary TB10-specific CD8^+^ T cells (Fig 7E). Of these unique sequences, 30% were also detected in the blood, and the remaining 70% were detected only in the lung (Fig 7F). Many of the clonotypes unique to the lung compartment were infrequent and in aggregate accounted for only 10% of the total T cells. Thus, 90% of the TB10-specific CD8^+^ T cells found in the lungs of infected mice used TCRs that were detected both in blood and in lung (Fig 7F). In fact, if only the highly abundant clonotypes (>0.5%) are considered, more than 99% of pulmonary TB10-specific CD8^+^ T cells use a TCR that is detected in peripheral blood during infection. Thus, clonotypes that are abundant and clonally expanded in the lung are also detected in peripheral blood.

### Selection drives T cell expansion following Mtb infection

**Fig 8:**
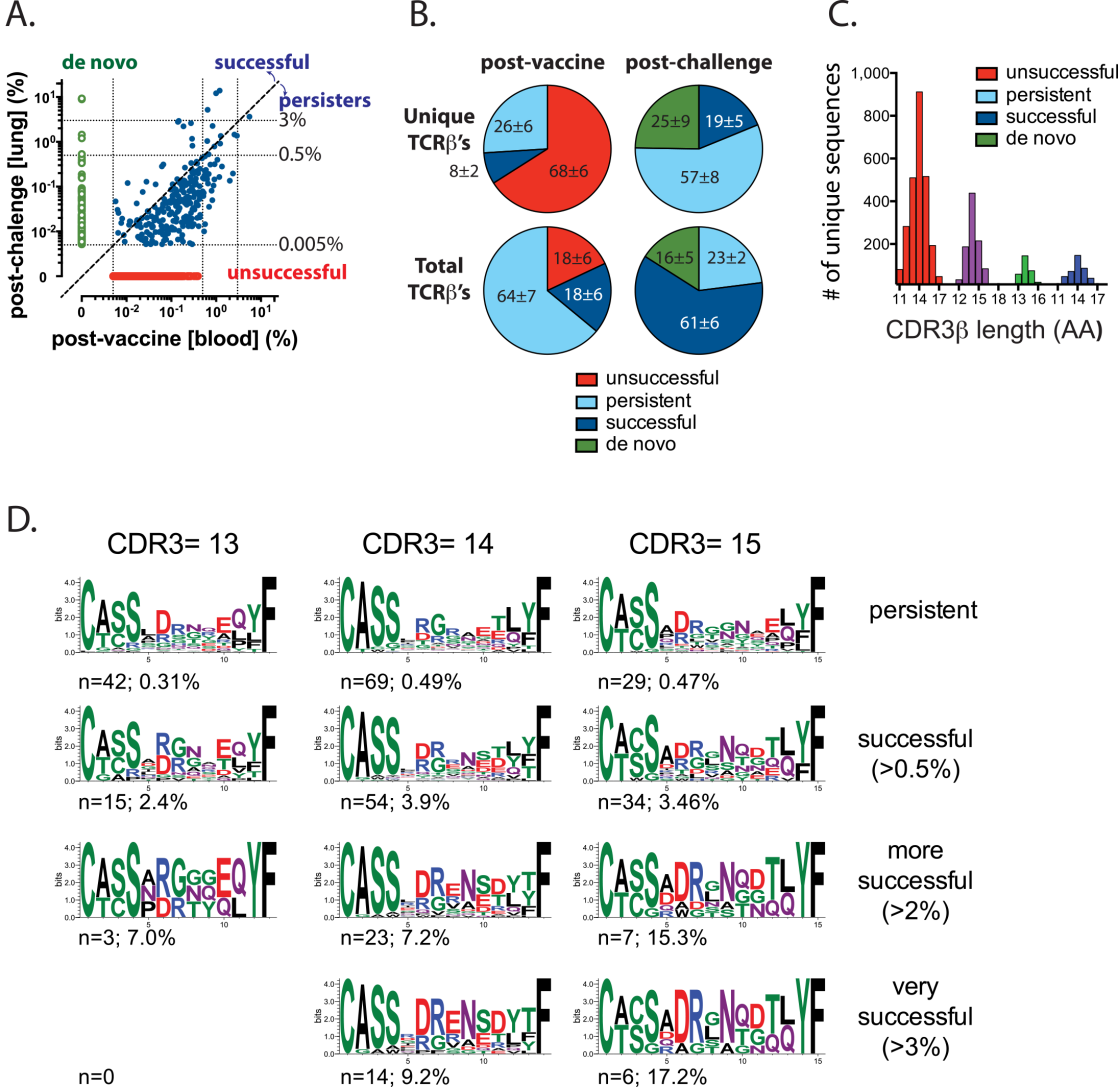
Selection drives the expansion of TB10-specific CD8^+^ T cells. **(a)** Representative logarithmic scatter plot showing the frequencies of all clonotypes detected in the post-vaccination (blood) or in the post-Mtb challenge (lung) repertoire of TB10-specific CD8^+^ T cells. Green, lung only (de novo response); red, blood only (unsuccessful); blue, shared (persistent & successful). The diagonal dashed line separates the successful and the persistent clonotypes. The dotted lines indicate frequencies of 0.005%, 0.5%, and 3%. **(b)** The proportion of total TCRβ amino acid sequences categorized as “unsuccessful”, “persisters”, “successful”, or “de novo” as defined in the text, from the post-vaccination (blood, left) or post-Mtb challenge (lung, right) repertoires. Top row, unique clonotypes; bottom row, total TCRs. Only clonotypes with a frequency of >0.005% were analyzed. **(c)** CDR3β length distribution among unique clonotypes categorized as “unsuccessful”, “persisters”, “successful”, or “de novo”. **(d)** CDR3β amino acid motifs were determined for highly prevalent clonotypes (>0.5% in post-vaccination or post-Mtb challenge repertoire), which were identified as “persisters” or “successful” TCR clonotypes with a CDR3 length of 13, 14, or 15 aa. For successful TCRs, different frequency thresholds were chosen (0.5%, 2%, or 3%) to identify structural motifs among highly prevalent clonotypes. The numbers below each sequence refer to the number of unique clonotypes that were used to derive the motif and the average frequency of each clonotype among total productive sequences.

To compare the fitness of each TCR clonotype responding to TB10.4 after Mtb challenge, we analyzed matched blood (post-vaccination) and lung (post-Mtb challenge) samples from individual subjects (S1 Table). Each unique TCRβ DNA sequence detected in the post-vaccination and post-challenge repertoire of the same individual were classified as “**successful**” if they increased in frequency after Mtb challenge or “**persisters**” if they decreased, but remained present post-challenge. Clonotypes were stratified as “**de novo**” or “**unsuccessful**” if they were present only after Mtb challenge or vaccination, respectively (Fig 8A). Interestingly, the two-thirds of the unique clonotypes elicited by vaccination are “unsuccessful” and are not detected after infection (Fig 8B). Although this is a large number of unique TCR sequences, they represent only ~18% of the total TB10-specific CD8^+^ T cell response in peripheral blood (Fig 8B). Conversely, after Mtb challenge, 25% of the unique clonotypes in the lung were de novo sequences, which accounted for ~16% of the total TB10-specific CD8^+^ T cells in lung (Fig 8B). Although each of these TCRs generally had a frequency of <0.5%, occasionally they were at a higher abundance (see Fig 8A as an example). Thus, although the naive T cells have the potential to expand more than memory T cells (Fig 4E and 4F, and [7,51,53,54]) and make a definable contribution to the recall response to Mtb infection, the majority of the total TCRs responding to Mtb challenge are a clonal population of 2° effectors derived from a relatively small number of “successful” vaccine-elicited T cells (Fig 8B). Finally, the majority of the vaccine-elicited T cells (67%) fail to expand during Mtb challenge, becoming “persisters” (Fig 8B). The fates of these “successful” and “persister” TCRs mirror the observed functions of the higher-affinity TB10Rg4 and lower-affinity TB10Rg3 CD8^+^ T cells, respectively, in our adoptive transfer studies (Fig 8B and Fig 6A and 6B).

Our clonality data suggested that infection was driving greater selection than vaccination. To identify structural features that govern TCR success or failure, we analyzed the CDR3β amino acid sequence of “**persisters**” and “**successful**” TCRβ clonotypes detected in the post-vaccination and post-challenge repertoire of the same individual. The CDR3β length distribution was similar among these four groups, with 14 amino acids being the most common length (Fig 8C). To focus on selection, we analyzed clonotypes that were present in both the post-vaccination and post-Mtb challenge repertoire, with a frequency of >0.5% in one of the samples. In addition, we restricted our analysis to clonotypes with a CDR3β length of 13, 14, or 15, which accounted for most of the sequences (Fig 8C). We identified 241 sequences from 7 individual subjects that met these criteria. These highly-represented clonotypes accounted for 60% of the productive sequences detected in the lungs of mice after Mtb challenge. Analysis showed that the “successful” clonotypes had a “DRxN” CDR3β motif (Fig 8D). The “DRENSD” motif, which had previously been detected among TB10-specific CD8^+^ T cells after Mtb infection in the absence of vaccination [32], was expressed by the most successful clones (Fig 8D). In contrast, “persister” clones lacked the “DRENSD” motif and instead more frequently encoded “RG” (Fig 8D). A similar motif was identified among those clonotypes with a CDR3β length of 15 amino acids. The persisters had a motif of “DRggNx” while the successful clones had a motif of “DRgNQD” (Fig 8D). These data indicate that during Mtb infection, selective pressure constrains the structural features of the TCR repertoire that recognize TB10.

## Discussion

It is unclear why vaccinated individuals, or those with prior Mtb infection, do not reliably exhibit protection from Mtb reinfection [14,55,56]. An often-cited benefit of recall immunity is its speed compared to naïve T cell responses, as shown during acute viral infection [57], homeostatic proliferation [58] and sterile antigen stimulation [59]. Both memory and naïve CD8^+^ T cells have been shown to expand equally well early during acute inflammation and in the presence of abundant antigen [51,53]. However, the contribution of memory T cells may decline under certain circumstances [51,53,54]. West et al find that memory CD8^+^ T cells proliferate poorly during a model of chronic infection (LCMV clone 13), possibly because persistently high antigen loads induce T cell exhaustion [7]. Mehlhop-Williams and Bevan find that following vaccination with immune-complexed ovalbumin (OVA), a higher antigen threshold is required to trigger proliferation of memory T cells than naïve T cells [51]. Finally, while memory OT-1 cells outperform naïve OT-1 cells after infection with *Listeria monocytogenes* expressing OVA; naïve OT-1 cells dominated after infection with *Plasmodium berghei* ANKA expressing OVA [54]. These studies raise the possibility that T cell vaccination could fail if the environment during infection hinders the expansion of memory T cells, which could minimize their contribution. A common thread in these studies is that memory T cells are disadvantaged when antigen is limiting, particularly during states of persistent inflammation. One limitation in these studies, however, is the use of OT-I cells and microbes engineered to over-express OVA rather than T cells specific for native microbial antigens. Our study is the first to evaluate the behavior of memory CD8^+^ T cells specific for a native immunodominant Mtb antigen using TCR retrogenic CD8^+^ T cells during infection. Furthermore, using peptide-challenge in two systems we show that the relative expansion of naïve and memory CD8^+^ T cells is determined by the amount of antigen present in their environment. Given the difficulty of developing a vaccine for tuberculosis, the fate of Mtb-specific memory T cells during challenge is an important question. We have shown that TB10-specific CD8^+^ T cells are under extreme selection and clonal expansions emerge even early during infection, which we infer is driven by a paucity of antigen presentation and the selection of high-affinity T cells [32]. We hypothesize that these same conditions could lead to memory T cell dysfunction during Mtb challenge.

Early initiation and sustained proliferation of a memory recall response are two characteristics that could affect the success of memory T cells during Mtb infection. We previously hypothesized that delayed initiation of T cell immunity is associated with susceptibility to Mtb [60]. Indeed, even a transient delay in T cell priming impairs control of Mtb in the lung [61]. Conversely, vaccinating C3H mice, whose adaptive immune response is delayed by ~1 week compared to C57BL/6 mice, with a DNA vaccine promoted an early recall response and CD8^+^ T cell-mediated reduction in bacterial CFU [62]. CD8^+^ T cell vaccination in CD4^-/-^ mice also led to protection after Mtb challenge [63]. In contrast, C57BL/6 mice have a more rapid 1° adaptive immune response and CD8^+^ T cell vaccination did not reduce bacterial CFU after Mtb challenge despite a robust memory response to vaccination [[33] and S1 Supporting Information]. These studies highlight the potential benefits of an early T cell response in controlling Mtb growth; however, the characteristics of memory T cells important for successful expansion during their response to Mtb have not yet been evaluated.

In this study, we discovered unexpected limitations in the expansion of memory-derived CD8^+^ T cells specific for an immunodominant Mtb antigen, providing one explanation for why T cell vaccines may be ineffective in preventing TB. By directly comparing naïve and memory CD8^+^ T cells using our adoptive co-transfer model, we show that both memory and naïve T cell responses are initiated in the MLN with similar kinetics. Thus, both primary and secondary responses are subject to significant delay before T cell expansion and recruitment to the lung occurs following Mtb challenge. Furthermore, once T cells traffic to the lungs, 2° effectors derived from memory precursors become rapidly outnumbered as their expansion plateaus after d15 post-infection, making them difficult to detect by d21. While this effect is modulated by TCR affinity (see below), these kinetics may explain why the superiority of natural memory T cell responses are limited to a narrow window early after infection [15,16]. Furthermore, the phenotype of the memory T cells we generated by vaccination is that of central memory (CD62L^Hi^ IL-7R^Hi^). As central memory T cells reside mostly in the draining lymph nodes, it may not be surprising that their activation occurs in the LN and requires trafficking of antigen and/or antigen-laden APCs. Although central memory T cells may be superior in mediating protection in adoptive transfer models, the requirement for priming in the LN could delay their response to Mtb, hindering the oft-cited benefit of a memory response: rapid recall.

Here, we show the crucial contribution of TCR affinity to the successful expansion of the 2° effector CD8^+^ T cells. In our adoptive co-transfer experiments, the response of memory TB10Rg3 and naïve TB10Rg3 CD8^+^ T cells results in domination by the 1° effectors by d21. As naïve and memory TB10Rg3 CD8^+^ T cells use an identical TCR and were co-transferred at a 1:1 ratio, factors other than TCR affinity or precursor frequency must affect T cell fitness after activation. However, there is enormous TCR diversity in individuals with intact immune systems and the success of individual clonotypes can be influenced by TCR affinity, particularly in environments characterized by little antigen presentation. Thus, in co-transfer experiments using the higher-affinity memory TB10Rg4 and naïve TB10Rg4 CD8^+^ T cells, we also observed skewing in favor of the 1° effector response, but the effect was much smaller. The role of TCR affinity is also demonstrated by co-transfer experiments using the higher-affinity memory TB10Rg4 CD8^+^ T cells and the lower-affinity naïve TB10Rg3 CD8^+^ T cells. Now we observe the opposite result: the memory TB10Rg4 cells (which have a higher TCR affinity) dominate the response by d21. Importantly, both TB10Rg3 and TB10Rg4 were dominant clones isolated from different individuals after aerosol Mtb infection [32]. Thus, eliciting memory T cells with high affinity for pMHC may make them more likely to successfully compete and expand during Mtb challenge. During natural infection in vaccinated individuals, competition between memory and naïve T cells of differing affinities is expected to occur; therefore, increasing the affinity of memory T cells should improve their fitness.

An important property of T cell memory responses is the increase in the number of T cells specific for the eliciting antigen [64]. Thus, the speed of the recall response is based in part on the greater precursor frequency of T cells that recognize the antigen challenge, independent of any increase in proliferative rate. By adjusting the ratio of naïve to memory T cells to a 1:1 ratio in our adoptive transfer studies, we control for any effect of T cell frequency. In contrast, our TCR sequencing of TB10 tetramer^+^ T cells from intact mice, post-vaccination and post-challenge, addresses differences between the 1° and 2° T cell responses in individuals in which both the avidity and abundance of individual clonotypes is allowed to vary. The majority of unique TCR clonotypes detected in the lungs of infected mice were initially detected post-vaccination. Interestingly, the dominant clonotypes were all derived from a narrow repertoire, suggesting that they were undergoing selection based on their affinity or other structural features during the response to Mtb infection. One such structural feature is the dominant TCRβ motif “DRENSD” that we find among TB10-specific CD8^+^ T cells in the lungs post Mtb-challenge (in our vaccinated mice), and is the same motif we previously identified in unvaccinated Mtb-infected mice [32], representing a highly-clonal response to Mtb. Thus, this motif appears to be highly-selected and independent of vaccination. We have shown that TB10-specific CD8^+^ T cells undergo massive clonal expansion in Mtb-infected mice, characterized by selection of the CDR3β motif, “DRENSD,” most likely driven by avidity for the TB10.4_4−11_/K^b^ complex, a hypothesis that we are preparing to test directly [32,65]. Interestingly, the majority of the clonotypes in the blood after vaccination were “persisters”, in that they did not increase in frequency relative to their abundance 1w post-vaccination in blood. Thus, the majority of vaccine-elicited TB10_4-11_-specific CD8^+^ T cells persist without expanding significantly during tuberculosis, and we relate this phenomenon to that observed after adoptive transfer of memory TB10Rg3 (lower affinity) CD8^+^ T cells into Mtb-infected mice, which are impaired in their expansion during TB. However, the higher-affinity TCR Rg CD8^+^ T cells (TB10Rg4) could be compared to a “successful” clonotypes from the TCR sequencing data as they displayed improved expansion in response to Mtb infection.

We used a peptide/CD40/poly(I:C) vaccination strategy that induces protection against viral infection and promotes tumor eradication [36,37,66]. The memory CD8^+^ T cells produced by using this vaccine strategy for TB10.4 were potent cytolytic effectors and cytokine-producers, and were able to attenuate infection when transferred to immunocompromised mice. Two other CD8^+^ T cell vaccines aimed at individual Mtb epitopes have also lowered bacterial burdens in intact mice, although the vaccinated hosts had a delayed adaptive immune response [62] or were deplete of CD4^+^ T cells [63]. A similarly potent vaccination strategy that elicits TB10-specific CD8^+^ T cells failed to protect intact mice against Mtb challenge [33]. In no TB vaccines, CD4 or CD8-focused, however, do we observe continued decline of bacterial CFU in the lungs, indicative of continuously functional T cells. Lindenstrøm et al attribute the lack of protection to limited presentation of the TB10.4_4−11_ epitope by infected cells during priming due to inefficient proteolytic cleavage of the TB10.4_4−11_ epitope [33]. Limited antigen presentation by infected cells could explain why TB10-specific CD8^+^ T cells undergo selection and clonal expansion and could be detrimental for memory T cell responses in tuberculosis. First, it is unknown whether current vaccine strategies elicit T cells of sufficient avidity to recognize the sparse antigen presented by infected cells. Second, memory T cells generated by vaccination may drop out of the response to infection if they require a higher antigen threshold for activation, independent of TCR affinity [51]. Why are class I MHC antigens poorly presented? In the case of TB10.4, the abundance of the protein in infected cells may be limiting. Alternatively, Mtb infection may inhibit class I MHC antigen presentation or infected macrophages in the lung may not be able to cross-present Mtb antigens. Remarkably, there exists little published data showing the direct recognition of Mtb-infected macrophages by CD8^+^ T cells [67,68]. Transfer of activated TB10Rg3 and TB10Rg4 CD8^+^ T cells to immunocompromised mice confers protection against Mtb in a TAP1-dependent manner [32]. We conclude from these data that infected macrophages present the TB10.4_4−11_ epitope in vivo. Although our current data demonstrate TCR selection, whether TCR avidity affects recognition of Mtb-infected macrophages and bacterial killing remain to be determined.

An important question is whether this idea of a low antigen state can be generalized to other antigens. We previously studied memory CD8^+^ T cells elicited by vaccination with a recombinant vaccinia virus (rVV, strain WR) expressing the Mtb antigen, EspA (Rv3616) or TB10.4 in BALB/c mice [69]. Two weeks after Mtb challenge, the numbers of EspA- and TB10.4-specific CD8^+^ T cells were significantly greater in the lungs of vaccinated mice compared to control mice, indicating a successful recall response. However, there were no differences in the numbers of TB10.4- or EspA-specific CD8^+^ T cells in vaccinated vs. control mice four weeks after Mtb challenge. Thus, despite using a different vaccination strategy (rVV) and different antigens (EspA, TB10.4), we observe similar results, namely that memory CD8^+^ T cell responses are less fit than primary CD8^+^ T cell responses. Furthermore, the decreased fitness of TB10.4-specific memory CD8^+^ T cells is not solely a consequence of the peptide/CD40/poly(I:C) vaccination strategy. The limited antigen recognition by Ag85b-specific CD4^+^ T cells [70–72] and the transient benefit of antibiotic-induced memory [15,16] suggest relevance beyond CD8^+^ T cells.

As memory T cells are a potent arm of adaptive immunity, impairing memory T cell function becomes a plausible step in the bacterium’s evolution as a pathogen. This complicates vaccine development against TB, as a successful candidate may need to generate high affinity T cells in order to compete with naïve, or vaccine-elicited, lower affinity T cells during TB. If a vaccine were to preferentially stimulate high affinity T cells, we predict that such T cells would be more fit during Mtb challenge. Although some argue that generating central memory T cells should be the goal of vaccination against Mtb [73,74], we and others argue that generating resident effector memory cells may be more important [3,75–77], as CD8^+^ T cells residing at the site of infection may be poised to initiate an earlier response. Screening known Mtb antigens for their ability to induce early memory T cell expansion during infection, and focusing on vaccines that generate high affinity T cells specific for those antigens could be an important next step in rational TB vaccine design.

## Materials and Methods

### Ethics statement

The animal studies were approved by the Institutional Animal Care and Use Committee at the Dana Farber Cancer Institute or the University of Massachusetts Medical School (Animal Welfare Assurance no. A3023-01 [DFCI] or A3306-01 [UMMS]), using the recommendations from the Guide for the Care and Use of Laboratory Animals of the National Institutes of Health and the Office of Laboratory Animal Welfare.

### Mice

C57BL/6J (WT; CD45.2^+^Thy1.2^+^), CD45.1 (B6.SJL-Ptprc^a^Pepc^b^/BoyJ; CD45.1^+^Thy1.2^+^), CD90.1 (B6.PL-Thy1^a^/CyJ; CD45.2^+^Thy1.1^+^), TCRα KO (B6.129S2-Tcra^tm1Mom^/J) mice were purchased from Jackson Laboratories (Bar Harbor, ME) and housed under specific pathogen-free conditions at Dana Farber Cancer Institute or University of Massachusetts Medical School animal facilities. Mice were 7 to 10 weeks old at the start of all experiments. Infected mice were housed in biosafety level 3 facilities under specific pathogen-free conditions at DFCI or UMMS.

### Generation of TCR retrogenic mice

TCR retroviral constructs were generated and retrogenic mice produced using protocols developed by the Vignali lab [78]. Details of the TCRs, cloning strategies and primer sequences have been recently published [32]. Retroviral-mediated stem cell gene transfer was performed using bone marrow from CD45.2^+^Thy1.2^+^, CD45.1^+^Thy1.2^+^, or CD45.2^+^Thy1.1^+^ mice, which was transferred into C57BL/6 recipients that were lethally-irradiated one day earlier with a split dose of 1200 Rads administered using a GammaCell 40 Cs^137^ Irradiator (Theratronics, Ottawa, ON, Canada). Reconstitution was measured 6 weeks later.

### Vaccination and assessment of immune responses

TB10.4_4−11_ (IMYNYPAM), B8R_20-27_ (TSYKFESV), “amphi-TB10” (MFVMFVQIMYNYPAM), and ovalbumin_257-264_ (SIINFEKL) peptides were purchased from New England Peptides (Gardner, MA, USA) and reconstituted in DMSO (10mM). High molecular weight polyinosinic:polycytidylic acid [poly(I:C)] was obtained from InvivoGen (San Diego, CA). Anti-CD40 mAb (clone FGK4.5) was purchased from BioXCell (West Lebanon, NH). Vaccines were prepared by mixing 100 micromoles of peptide, 50 μg poly(I:C), and 50 μg αCD40 mAb, in a total volume of 200 μL sterile PBS and administered intravenously. Where indicated, mice were boosted with the same vaccine 3 weeks later. In some experiments, peripheral blood T cell responses were monitored by flow cytometry. Mice were rested 8–12 weeks after the last vaccination to allow for the development of memory. Memory cells were generated using two different strategies. Since serial adoptive transfers of memory T cells can decrease their protective and proliferative capacities [79], TB10Rg3 or TB10Rg4 mice having a low frequency of peripheral blood retrogenic cells (3–17%), were directly vaccinated with TB10/CD40/poly(I:C). In other experiments, 20,000 naïve TB10Rg3 CD8^+^ T cells were adoptively transferred into C57BL/6 mice (resulting in 200–2,000 naïve precursor T cells after a 1–10% “take”). Those mice were then vaccinated as described above. In both cases, mice were rested for 8–12 weeks after vaccination to allow the development of memory. A comparison of memory TB10Rg3 CD8^+^ T cells elicited by vaccination after adoptive transfer of TB10Rg3 cells into B6 mice to those generated by vaccination of intact retrogenic mice showed similar results. Naïve TB10-specific CD8^+^ T cells were obtained from unvaccinated, age-matched TB10Rg3 or TB10Rg4 mice rested for an equivalent period of time.

### Experimental infection and bacterial quantification

Mtb (strain Erdman) infections were performed via the aerosol route as described previously [60]. Infections at UMMS were performed using a Glas-Col full body inhalation exposure system (Terre Haute, IN). Mice received an inoculation dose of 30–190 CFU/mouse, as measured within 24 hours of infection. At different times post-infection, mice were euthanized, organs were aseptically removed, individually homogenized, and viable bacteria were enumerated by plating 10-fold serial dilutions of organ homogenates onto 7H11 agar plates. Recombinant *Listeria monocytogenes* expressing the full-length TB10.4 coding sequence (LmΔActA-TB10) was generated by amplifying the full-length coding sequence of TB10.4 from Mtb genomic DNA and cloning into the gram positive expression vector pAM401 behind the promoter and signal sequence for *Listeria monocytogenes hly* encoding Listeriolysin-O. This construct was electroporated into attenuated ΔActA *Listeria monocytogenes* that retains access to the cytoplasmic compartment of infected cells as described previously for ESAT-6 and Ag85b constructs [49,50]. Bacteria containing the plasmid were grown to mid-log phase (OD_600_ 0.4–0.8) in brain-heart infusion media (BHI) (Sigma) supplemented with chloramphenicol (10μg/mL) (Sigma) and aliquots were frozen at -80°C. Experiments using LmΔActA-TB10 were performed by injecting host mice with 10^7^ bacteria 1 day after 1:1 co-transfer of 10^4^ naïve and memory TB10Rg3 cells. Bacterial titers were enumerated by plating 10-fold serial dilutions of inoculum onto BHI agarose supplemented with chloramphenicol (10μg/mL).

### Flow cytometric analysis

Lungs, spleen, and LNs were removed after lung perfusion with 10mL of cold RPMI1640. Lung cell suspensions were prepared by coarse dissociation using the GentleMACS tissue dissociator (Miltenyi Biotec, Germany). Tissue was digested for 30–60 min at 37°C with 250 U/mL collagenase (Sigma) in complete RPMI1640 [10% heat inactivated FCS (Sigma), 10 mM HEPES, 1 mM sodium pyruvate, 2 mM L-glutamine, 10mM β-mercaptoethanol, 50 mg/ml streptomycin and 50 U/ml penicillin (all from Invitrogen)] followed by homogenization in the GentleMACS dissociator and sequential straining through 70 μm and 40 μm nylon cell strainers (Falcon). Spleen and LN cell suspensions were prepared using gentle disruption of the organs through a 70 μm nylon strainer, followed by a 40 μm nylon cell strainer. For some experiments, erythrocytes were lysed in using a hemolytic solution. For co-transfer experiments using naïve and memory TCR Rg CD8^+^ T cells, CD8^+^ T cells were enriched prior to surface antibody staining using either positive or negative selection (Mouse CD8 T cell isolation kit or CD8 T cell isolation kit II, Miltenyi Biotec). Surface staining was performed with antibodies specific for mouse CD3ε (clone 145-2C11), CD4 (clone GK1.5), CD8 (clone 53–6.7), CD19 (clone 6D5), CD44 (clone IM7), CD62L (clone MEL-14), CD127 (clone A7R34), KLRG1 (clone 2F1/KLRG1), CXCR3 (clone CXCR3-173), CD45.1 (clone A20), CD45.2 (clone 104), CD90.1 (clone OX-7), CD90.2 (clone 53–2.1), Vα2 (clone B20.1), Vβ11 (clone KT11) (all from Biolegend, CA, USA). TB10.4_4–11_-loaded and B8R_20-27_-loaded H-2K^b^ tetramers were obtained from the National Institutes of Health Tetramer Core Facility (Emory University Vaccine Center, Atlanta, GA, USA). For most experiments, duel-tetramer staining was performed as described [80] using PE- and APC-conjugated TB10.4_4–11_-loaded tetramers together to accurately enumerate low-frequency events and minimize false-positive signal. For adoptive co-transfer experiments, an amine-reactive viability dye, Zombie Aqua (Biolegend) was used to exclude necrotic cells. The active caspase-3 apoptosis antibody kit was measure apoptosis (clone C92-605, Beckton Dickinson, CA). All samples from Mtb-infected mice were fixed with 1% paraformaldehyde before analysis. Data were acquired using a FACS Canto II (Becton Dickinson) or a MACSQuant flow cytometer (Miltenyi Biotec). Data were analyzed using FlowJo Software V9 (Tree Star, OR). For both analysis and cell sorting, single-lymphocyte events were gated by forward scatter area and height versus side scatter area for size and granularity.

### Adoptive T cell transfer of CD8^+^ T cells

Single cell suspensions of homogenized spleens and LNs (inguinal, cervical, axillary, mediastinal, and mesenteric) were prepared from vaccinated retrogenic mice (8–12 weeks after the last vaccination) or age-matched unvaccinated mice. CD8^+^ T cells were purified by negative selection using the CD8^+^ T cell isolation kit II (Miltenyi Biotec) or the EasySep mouse CD8 T cell enrichment kit (StemCell Technologies, Vancouver, BC, Canada) followed by magnetic separation. After purification, cells were stained with eFluor 450 proliferation dye (eBiosciences), antibody-stained and sorted by flow cytometry to achieve uniform populations of naïve or memory CD8^+^ T cells. For TB10Rg3 naïve/memory co-transfer experiments, 1x10^4^ cells of each population were mixed at a 1:1 ratio (confirmed by flow cytometry) and were transferred IV into congenic recipients (CD90.1 or CD45.1), which had been infected 0–7 d earlier with Mtb. TB10Rg3 CD8^+^ T cells used for the memory group were generated on the Thy1.1^+^, CD45.1^+^, and Thy1.2^+^CD45.2^+^ backgrounds to ensure that none of the observed effects were specific to congenic backgrounds of the mice. For protection experiments, 1x10^5^ TB10Rg3 Thy1.2^+^CD45.2^+^ memory or naïve cells were transferred into TCRα^-/-^ mice or sub-lethally irradiated (600 Rads) C57BL/6 mice, and challenged with Mtb the same day.

### Cell sorting

Fluorescent antibody-stained cells were sorted using a FACS Aria II (Becton Dickinson) flow cytometer. For adoptive transfer experiments, CD8^+^CD4^-^GFP^+^Vα2^+^ KLRG1^Lo^ CD44^hi^ memory (from vaccinated Rg mice) or CD44^Lo^ naïve TB10Rg3 cells (from age-matched unvaccinated Rg mice) were sorted from pre-enriched CD8^+^ T cells. For TCRβ repertoire analysis, we used duel-tetramer staining to identify and sort CD8^+^CD4^-^tetramer^++^CD44^Hi^ T cells from blood, one week after vaccination with amphi-TB10/CD40/poly(I:C). Twelve weeks after vaccination, the same mice were infected with Mtb, and five weeks later, CD8^+^CD4^-^tetramer^++^CD44^Hi^ T cells were again sorted from the lungs.

### Next generation sequencing

Genomic DNA was purified from sorted TB10 tetramer^+^ CD44^+^CD8^+^ T cell populations using the QIAamp DNA Mini kit or QIAamp DNA FFPE Tissue kit (for fixed cells) (both from Qiagen). High-throughput TCRβ sequencing was performed by Adaptive Biotechnologies Corp. (Seattle, WA) (http://www.immunoseq.com) and analyzed using the ImmunoSEQ analyser toolset [81]. Clonality was calculated as entropy of the frequency distribution 1-(entropy/log_2_[# unique TCRs]). Entropy, a measure of diversity within a complex data set, is also known as the Shannon-Wiener index, Shannon’s diversity index or Shannon’s entropy [82,83]. Transforming entropy in this manner results in a clonality score on a scale between 0–1. A score of “0” indicates that every TCR is unique; a score of “1” means that every TCR is the same. WebLogo 3 was used to identify CDR3β motifs (http://weblogo.threeplusone.com). S6 Supporting Information identifies the different samples, their characteristics, and their inclusion in the different analyses and figures.

### Intracellular cytokine staining and ELISAs

Lung cells cultured with rhIL-2 (100 U/mL; Peprotech) were stimulated in the presence or absence of TB10.4_4−11_ peptide (10 μM; New England Peptides) or αCD3/αCD28 mAbs (1 mg/mL, BioLegend) as positive control. After 1 h at 37°C, GolgiStop (BD Pharmingen, CA, USA) was added for 4 h. Cells were then stained with antibodies, permeablized (BD Permwash Kit; BD Pharmingen), and stained for IFNγ (clone XMG1.2; Biolegend), TNF (clone MP6-XT22; Biolegend), or Granzyme B (clone GB11; Biolegend). For ELISAs, IFNγ, TNF, and IL-2 production were measured in supernatants after 48h stimulation with peptide, using ELISA Max standard kits (Biolegend).

### *In vivo* CTL assay

Cytotoxicity was determined using peptide-coated splenocytes from congenic CD45.1^+^ B6 mice as target cells, differentially-labeled with the cell proliferation dyes eFluor 450 (eBiosciences) and/or CFSE (eBiosciences) as previously described [29]. Briefly, target cells were pulsed with 10 μM, 1 μM, 0.33 μM, 0.1 μM, 0.033 μM, or 0.01 μM of TB10.4_4−11_ peptide, or left unpulsed (as control). Six of the target cell populations were then labeled with 5 μM, 1.25 μM, or 0.3125 μM eFluor 450 dye (two populations for each dye concentration). Prior to washing, three of the six populations (one for each of the eFluor 450 dye concentrations) were also labeled with 5 μM CFSE. The 7^th^ population of peptide-coated targets was stained only with 5 μM CFSE. After extensive washing, labeled populations were mixed at equal cell ratios and 3–5 x 10^6^ cells per population (21–35 x 10^6^ total cells) were injected into TB10/CD40/poly(I:C)-vaccinated or unvaccinated control CD45.2^+^ B6 mice. After 20h, the relative proportions of each populations in the lung and spleen were determined by flow cytometry and compared unvaccinated control mice as described [29].

### Measurement of cell proliferation

Analysis TB10Rg3 cell proliferation was measured after adoptive transfer into Mtb-infected, vaccinated, or TCRα^-/-^ mice, or *in vitro* after stimulation by labeling purified TB10Rg3 CD8^+^ T cells with 5 μM of the cell proliferation dye eFluor 450 (eBiosciences). Proliferation, as measured by dye dilution, was measured by flow cytometry *in vivo* 11d after aerosol Mtb infection, or *in vitro* 64h after co-culture with APCs coated with a serial dilution of TB10.4_4−11_ peptide. Cell proliferation at later time points (d15 or d18) *in vivo* was assayed by the incorporation of the synthetic thymidine analogue 5-Ethynyl-2’-deoxyuridine (EdU, Life Technologies). Briefly, 1mg EdU diluted in 100 μL PBS was injected i.p. into each mouse 12h prior to analysis. After antibody staining, single cells suspensions were assayed for EdU incorporation using the Click-iT EdU Alexa Fluor 647 Flow Cytometry Assay kit (Life Technologies).

### Statistical analysis

Data are represented as mean ± SEM. A two-tailed student’s t-test was used for normally-distributed data to compare two groups. One-way or Two way ANOVA were used to compare more than two groups, followed by Bonferroni or Sidak post-tests. A p value < 0.05 was considered to be statistically significant. Statistical analyses were performed using Prism V6 (GraphPad Software, San Diego, CA).

## Supporting Information

S1 Fig Vaccination with TB10.4_4−11_ does not protect mice against Mtb infection. **(a)** Lung CFU 14d and 28d after Mtb infection of TB10_4-11_ vaccinated, control vaccinated (B8R_20-27_ or Ova_257-264_), or unvaccinated mice. (**b**) Lung CFU 28d after Mtb infection of amphiphilic-TB10_4-11_ (amphi-TB10) or B8R_20-27_ vaccinated mice. Bacterial counts were log_10_-transformed and compared using a student’s t-test or one-way ANOVA. n.s., not significant. Data are representative of 3–6 independent experiments, each with 4–6 mice per group.
(PDF)

S2 Fig 1° and 2° TB10Rg3 CD8^+^ T cells contain equal expression of cell death markers. Bar graphs of the frequency of activated caspase-3 expression and viability dye Zombie Aqua (Biolegend) expression on TB10Rg3 cells derived from naïve (1°) and memory (2°) precursors in the lungs of Mtb-infected mice 15d post aerosol Mtb challenge. Two-way ANOVA with sidak post-test were used to compare marker expression in each group. n.s. not significant. Data are representative of 3 independent experiments, each with 3–4 mice per group.
(PDF)

S3 Fig Memory and Naïve T cells expand equally during homeostatic proliferation. **(a)** Histogram of proportions of memory (red) and naïve (blue) TB10Rg3 cells that have diluted proliferation dye eFluor450, recovered from the spleens 21d after adoptive-co-transfer into TCRα^-/-^ mice. **(b)** Bar graph comparing the ratios of naïve and memory TB10Rg3 cells (mean ± SEM) among those undergoing >1 division during homeostatic proliferation. Ratios were compared using student’s t-tests. n.s. not significant. Data are representative of 2 independent experiments, each with 3–4 mice per group.
(PDF)

S4 Fig 1° and 2° TB10Rg4 CD8^+^ T cells contain equal TCR expression. Bar graphs of median fluorescence intensity (MFI) of TCR Vα2 expression on TB10Rg4 cells derived from naïve (1°) and memory (2°) precursors in the lungs of Mtb-infected mice 14, 18, and 21d post aerosol Mtb challenge. Vα2 MFIs were compared using student’s t-tests for each time point. n.s. not significant. Data are representative of 2 independent experiments, each with 4 mice per group (time point).
(PDF)

S5 Fig TCR analysis of mice vaccinated with TB10.4_4−11_ and challenged with Mtb. For two representative mice (mouse #2 and #4), the TCR repertoire of TB10.4_4−11_-specific CD8^+^ T cells is presented 1 week after they received a vaccine boost (top row) and 5 weeks after they were challenged with virulent Mtb (bottom row). The post-vaccine repertoire was obtained from TB10.4_4−11_-specific CD8^+^ T cells purified from peripheral blood and the post-Mtb challenge repertoire was obtained from cells purified from the lung. The clonality index is shown for each repertoire. As discussed in the text, the clonality of the TB10.4_4−11_-specific CD8^+^ T cell repertoire increases after Mtb infection. The TCR repertoire is plotted as frequency (Z-axis) of each Vβ gene (X-axis) and CDR3β length combination.
(PDF)

S1 Table Samples analyzed by NexGen sequencing. Adaptive Inc. performed deep TCRβ sequencing using the ImmunoSeq assay. In the first experiment, samples 1–6 were obtained from blood one week after vaccination; samples 7–12 were obtained from lung 5 weeks after Mtb challenge. In the second experiment, samples 13–16 were obtained from blood one week after vaccination; samples 17–20 and 21–24 were obtained from blood and lung, respectively, 4 weeks after Mtb challenge. All samples were DNA from flow sorted TB10-specific CD8^+^ T cells (as described in the methods). The summary data for each sample is shown including the total number of reads, the unique number of reads, the productive total and unique reads, the clonality, the mass of the reaction template, the maximum frequency, and the total number of gene rearrangements, and the depth of coverage. The level of sequencing (survey vs. deep) is also indicated. Various samples were used for the analyses shown in the figures. Inclusion in the analysis is designated by a checkmark. Some samples were specifically excluded from an analysis (designated by an ‘x’). If a sample was excluded, the reason is listed.
(PDF)
