# Supplementary material for: A Higher Activation Threshold of Memory CD8+ T Cells Has a Fitness Cost That Is Modified by TCR Affinity during Tuberculosis
Source: PLoS Pathog. 2016 Jan 8;12(1):e1005380. doi: 10.1371/journal.ppat.1005380 (PMC4706326; doi:10.1371/journal.ppat.1005380)
Supplement: S1 Fig — (a) Lung CFU 14d and 28d after Mtb infection of TB104-11 vaccinated, control vaccinated (B8R20-27 or Ova257-264), or unvaccinated mice. (b) Lung CFU 28d after Mtb infection of amphiphilic-TB104-11 (amphi-TB10) or B8R20-27 vaccinated mice. Bacterial counts were log10-transformed and compared using a student’s t-test or one-way ANOVA. n.s., not significant. Data are representative of 3–6 independent experiments, each with 4–6 mice per group. (PDF) [file ppat.1005380.s001.pdf]

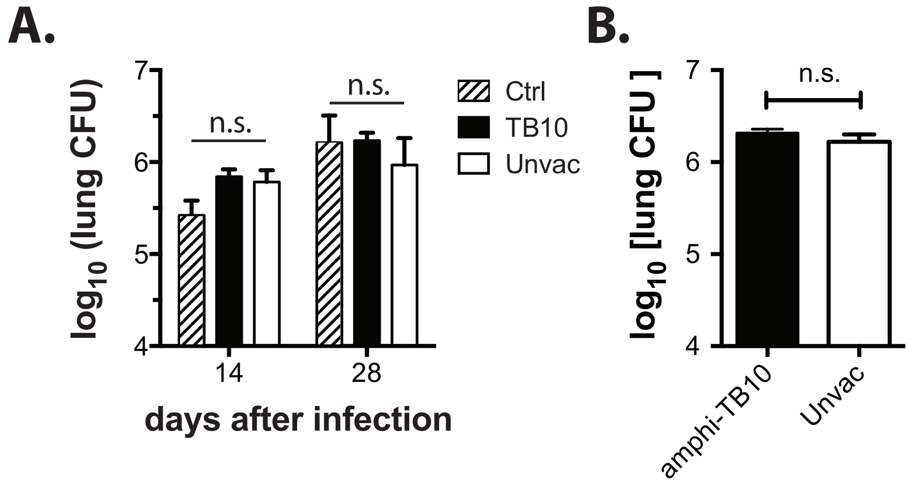

S1 Supporting Information:

Vaccination with TB10.4$_{4\text{-}11}$ does not protect mice against Mtb infection.
