# Supplementary material for: A Higher Activation Threshold of Memory CD8+ T Cells Has a Fitness Cost That Is Modified by TCR Affinity during Tuberculosis
Source: PLoS Pathog. 2016 Jan 8;12(1):e1005380. doi: 10.1371/journal.ppat.1005380 (PMC4706326; doi:10.1371/journal.ppat.1005380)
Supplement: S2 Fig — Bar graphs of the frequency of activated caspase-3 expression and viability dye Zombie Aqua (Biolegend) expression on TB10Rg3 cells derived from naïve (1°) and memory (2°) precursors in the lungs of Mtb-infected mice 15d post aerosol Mtb challenge. Two-way ANOVA with sidak post-test were used to compare marker expression in each group. n.s. not significant. Data are representative of 3 independent experiments, each with 3–4 mice per group. (PDF) [file ppat.1005380.s002.pdf]

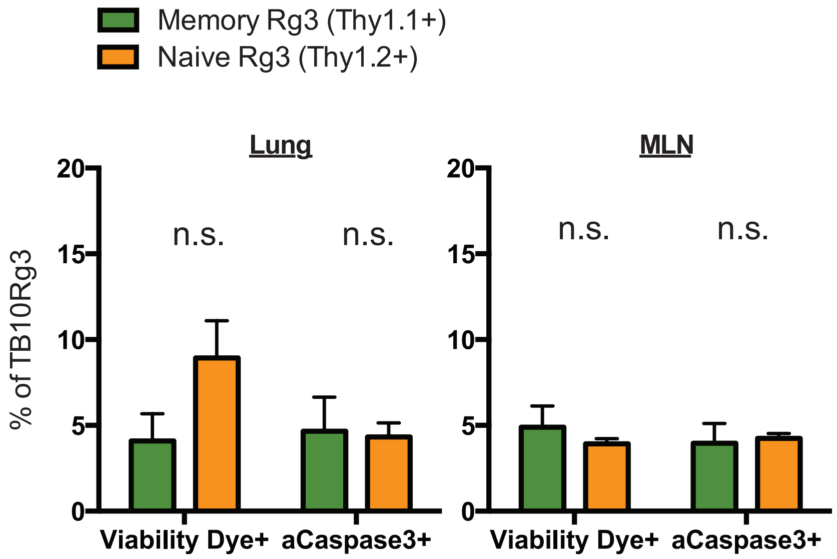

S2 Supporting Information:
1° and 2° TB10Rg3 $CD8^+$ T cells contain equal expression of cell death markers
