# Supplementary material for: A Higher Activation Threshold of Memory CD8+ T Cells Has a Fitness Cost That Is Modified by TCR Affinity during Tuberculosis
Source: PLoS Pathog. 2016 Jan 8;12(1):e1005380. doi: 10.1371/journal.ppat.1005380 (PMC4706326; doi:10.1371/journal.ppat.1005380)
Supplement: S3 Fig — (a) Histogram of proportions of memory (red) and naïve (blue) TB10Rg3 cells that have diluted proliferation dye eFluor450, recovered from the spleens 21d after adoptive-co-transfer into TCRα-/- mice. (b) Bar graph comparing the ratios of naïve and memory TB10Rg3 cells (mean ± SEM) among those undergoing >1 division during homeostatic proliferation. Ratios were compared using student’s t-tests. n.s. not significant. Data are representative of 2 independent experiments, each with 3–4 mice per group. (PDF) [file ppat.1005380.s003.pdf]

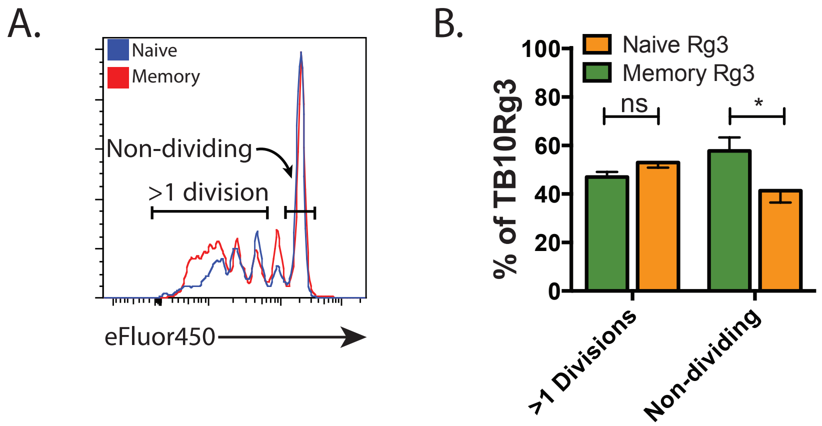

S3 Supporting Information:
Memory and Naïve T cells expand equally during homeostatic proliferation
