# Supplementary figures and images for: A Higher Activation Threshold of Memory CD8+ T Cells Has a Fitness Cost That Is Modified by TCR Affinity during Tuberculosis
Source: PLoS Pathog. 2016 Jan 8;12(1):e1005380. doi: 10.1371/journal.ppat.1005380 (PMC4706326; doi:10.1371/journal.ppat.1005380)

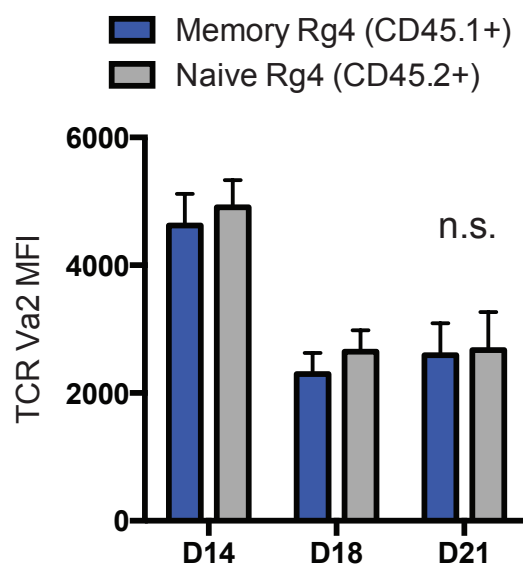

S4 Supporting Information:

1° and 2° TB10Rg4 CD8<sup>+</sup> T cells contain equal TCR expression

Supplement: S4 Fig — Bar graphs of median fluorescence intensity (MFI) of TCR Vα2 expression on TB10Rg4 cells derived from naïve (1°) and memory (2°) precursors in the lungs of Mtb-infected mice 14, 18, and 21d post aerosol Mtb challenge. Vα2 MFIs were compared using student’s t-tests for each time point. n.s. not significant. Data are representative of 2 independent experiments, each with 4 mice per group (time point). (PDF) [file ppat.1005380.s004.pdf]
