# Supplementary material for: A Higher Activation Threshold of Memory CD8+ T Cells Has a Fitness Cost That Is Modified by TCR Affinity during Tuberculosis
Source: PLoS Pathog. 2016 Jan 8;12(1):e1005380. doi: 10.1371/journal.ppat.1005380 (PMC4706326; doi:10.1371/journal.ppat.1005380)
Supplement: S5 Fig — For two representative mice (mouse #2 and #4), the TCR repertoire of TB10.44−11-specific CD8+ T cells is presented 1 week after they received a vaccine boost (top row) and 5 weeks after they were challenged with virulent Mtb (bottom row). The post-vaccine repertoire was obtained from TB10.44−11-specific CD8+ T cells purified from peripheral blood and the post-Mtb challenge repertoire was obtained from cells purified from the lung. The clonality index is shown for each repertoire. As discussed in the text, the clonality of the TB10.44−11-specific CD8+ T cell repertoire increases after Mtb infection. The TCR repertoire is plotted as frequency (Z-axis) of each Vβ gene (X-axis) and CDR3β length combination. (PDF) [file ppat.1005380.s005.pdf]

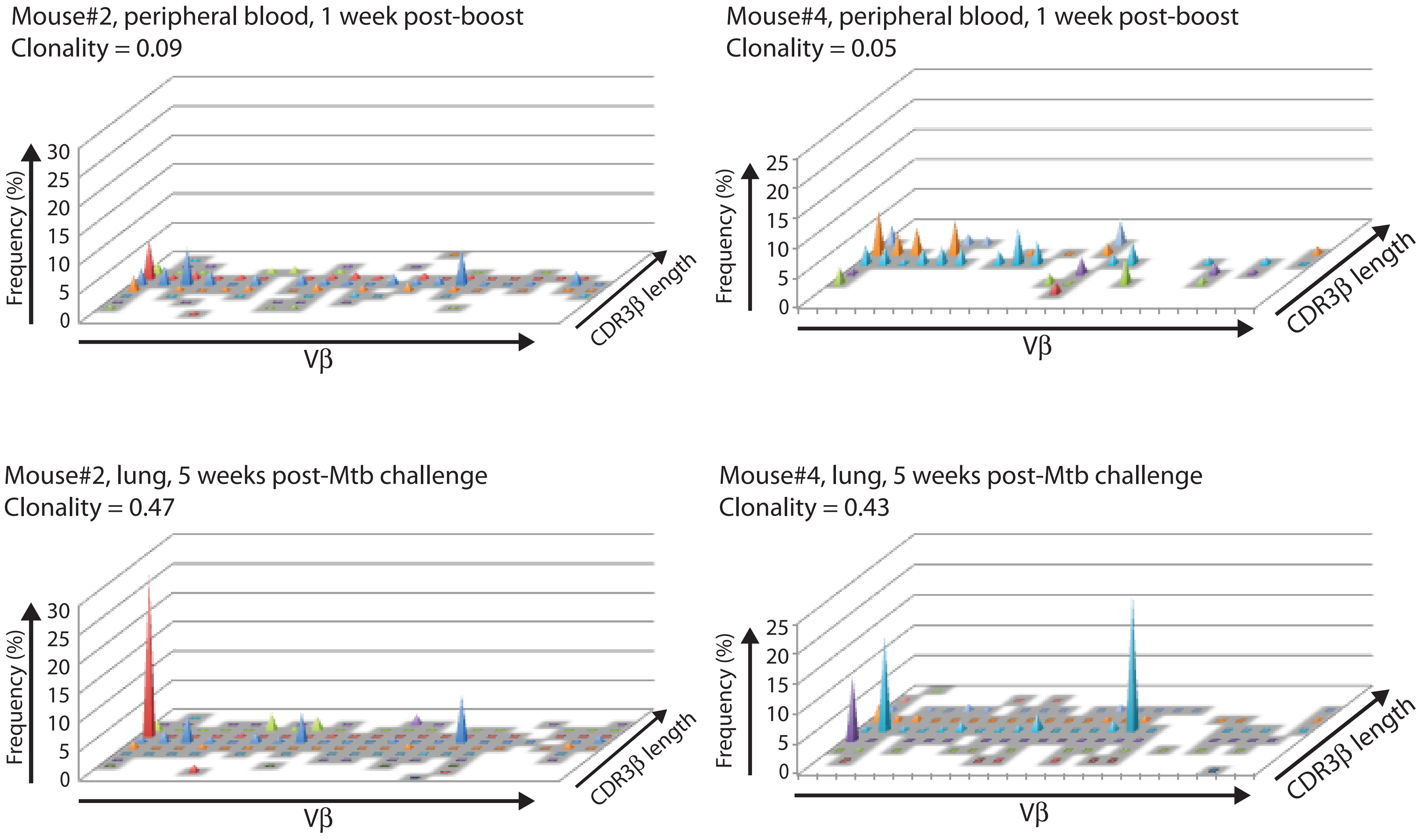

S5 Supporting Information:
TCR analysis of mice vaccinated with $TB10.4_{4-11}$ and challenged with Mtb
