# Supplementary material for: A Higher Activation Threshold of Memory CD8+ T Cells Has a Fitness Cost That Is Modified by TCR Affinity during Tuberculosis
Source: PLoS Pathog. 2016 Jan 8;12(1):e1005380. doi: 10.1371/journal.ppat.1005380 (PMC4706326; doi:10.1371/journal.ppat.1005380)
Supplement: S1 Table — Adaptive Inc. performed deep TCRβ sequencing using the ImmunoSeq assay. In the first experiment, samples 1–6 were obtained from blood one week after vaccination; samples 7–12 were obtained from lung 5 weeks after Mtb challenge. In the second experiment, samples 13–16 were obtained from blood one week after vaccination; samples 17–20 and 21–24 were obtained from blood and lung, respectively, 4 weeks after Mtb challenge. All samples were DNA from flow sorted TB10-specific CD8+ T cells (as described in the methods). The summary data for each sample is shown including the total number of reads, the unique number of reads, the productive total and unique reads, the clonality, the mass of the reaction template, the maximum frequency, and the total number of gene rearrangements, and the depth of coverage. The level of sequencing (survey vs. deep) is also indicated. Various samples were used for the analyses shown in the figures. Inclusion in the analysis is designated by a checkmark. Some samples were specifically excluded from an analysis (designated by an ‘x’). If a sample was excluded, the reason is listed. (PDF) [file ppat.1005380.s006.pdf]

SUPPLEMENTAL TABLE I

	Sample Name	Total	Unique	Productive Total	Productive Unique	Clonality	Mass of Reaction Template (ng)	Max Frequency (%)	Gene Rearrangements	Coverage	motifs	level	Fig7a	Fig7c	Fig7d	Fig7e	Fig1f	Fig8b	Fig8c	Fig8d	comment
1	B6TrivaxTB10_1w_1	36,110	359	25,947	253	0.133	84.2	4.3	869	51	✓	survey								✓	
2	B6TrivaxTB10_1w_2	21,551	331	15,423	231	0.091	66.9	2.9	479	58	✓	survey								✓	
3	B6TrivaxTB10_1w_3	5,290	80	3,818	55	0.079	78.7	5.0	95	77	✓	survey								✓	
4	B6TrivaxTB10_1w_4	4,298	70	3,863	58	0.051	51.8	4.4	83	85	✗	survey								✗	excluded because of too few sequences (blood)
5	B6TrivaxTB10_1w_5	6,747	156	4,801	110	0.110	112.6	6.2	200	43	✗	survey								✗	excluded because of too few sequences (blood)
6	B6TrivaxTB10_1w_6	104	12	54	6	0.070	73.6	16.3	12	14	✗	survey								✗	excluded because of too few sequences (blood)
7	B6TrivaxTB10_5wInf_1	1,366,057	957	1,183,681	624	0.528	364.8	38.2	27,873	56	✓	survey								✓	
8	B6TrivaxTB10_5wInf_2	1,529,724	1,252	1,137,552	793	0.471	400.0	13.5	27,449	57	✓	survey								✓	
9	B6TrivaxTB10_5wInf_3	1,418,738	677	1,018,085	454	0.557	400.0	22.1	38,190	40	✓	survey								✓	
10	B6TrivaxTB10_5wInf_4	491,958	632	375,641	397	0.431	321.0	17.0	6,719	75	✗	survey								✗	excluded because of too few sequences (from paired blood sample)
11	B6TrivaxTB10_5wInf_5	918,589	571	751,499	346	0.470	320.3	22.8	5,754	171	✗	survey								✗	excluded because of too few sequences (from paired blood sample)
12	B6TrivaxTB10_5wInf_6	101,521	309	78,746	204	0.411	283.5	20.1	5,408	20	✗	survey								✗	excluded because of too few sequences (from paired blood sample)
13	aTB10prime_B7	1,135,007	3,076	822,168	2,048	0.223	844.8	3.1	18,933	65	✓	deep	✓	✓	✓			✓	✓	✓	
14	aTB10prime_B8	1,533,180	4,091	1,099,553	2,675	0.245	1036.8	1.8	29,133	58	✓	deep	✓	✓	✓			✓	✓	✓	
15	aTB10prime_B9	1,605,354	4,110	1,160,134	2,776	0.226	965.8	1.6	25,362	70	✓	deep	✓	✓	✓			✓	✓	✓	
16	aTB10prime_B10	705,086	2,334	514,497	1,517	0.215	937.0	4.2	8,829	96	✓	deep	✓	✓	✓			✓	✓	✓	
17	Mtb4wTB10_B1	208,451	935	173,848	646	0.429	400.0	16.5	4,536	50		survey				✓	✓				
18	Mtb4wTB10_B2	150,387	799	107,173	562	0.353	400.0	14.2	2,958	57		survey				✓	✓				
19	Mtb4wTB10_B3	3,736	97	2,809	69	0.138	400.0	5.8	136	36	✗	survey				✗	✗				excluded because of too few sequences (blood)
20	Mtb4wTB10_B4	172,707	822	132,516	562	0.408	400.0	18.7	4,536	41		survey				✓	✓				
21	Mtb4wTB10_L1	747,497	1,260	586,252	828	0.405	400.0	19.5	17,300	45	✓	survey	✓	✓	✓	✓	✓	✓	✓	✓	
22	Mtb4wTB10_L2	545,130	1,049	401,203	690	0.300	400.0	6.3	13,048	43	✓	survey	✓	✓	✓	✓	✓	✓	✓	✓	
23	Mtb4wTB10_L3	127,013	682	101,008	486	0.309	400.0	10.0	2,766	50	✓	survey	✓	✓	✓	✗	✗	✓	✓	✓	excluded because of too few sequences (from paired blood sample)
24	Mtb4wTB10_L4	300,867	886	213,295	604	0.368	400.0	15.9	9,398	33	✓	survey	✓	✓	✓	✓	✓	✓	✓	✓	
